# Telephone-Based Training Intervention for Using Digital Communication Technologies for Social Housing Residents During the COVID-19 Pandemic: Mixed Methods Feasibility and Acceptability Evaluation

**DOI:** 10.2196/45506

**Published:** 2024-01-26

**Authors:** Tim Walker, Sarah Ann Buckingham, Ria Poole, Lewis Roland Elliott, Tamaryn Menneer, Gengyang Tu, Karyn Morrissey

**Affiliations:** 1 European Centre for Environment and Human Health University of Exeter Medical School Truro United Kingdom; 2 International Business School Suzhou Xi’an Jiaotong-Liverpool University Suzhou China; 3 Department of Technology, Management and Economics Technical University of Denmark Lyngby Denmark

**Keywords:** digital training, telephone-based, social housing, feasibility, acceptability, communication technologies, sociodigital inequalities, mobile phone

## Abstract

**Background:**

In an era in which digital communication technologies play a pivotal role in everyday life, social housing residents remain highly susceptible to digital exclusion.

**Objective:**

This study aims to evaluate the feasibility and acceptability of a telephone-based training intervention designed to empower people to confidently use digital communication technologies (ie, video calls and web-based messaging).

**Methods:**

Conducted in collaboration with a UK social housing association, the intervention was facilitated by a unitary authority’s Digital Inclusion Team during the COVID-19 pandemic. A mixed methods approach was used, encompassing quantitative and qualitative data collection on demand, reach, implementation, and potential outcomes. Demographic and qualitative data on the reasons for undertaking or not undertaking the training were collected via telephone interviews during the recruitment process. Digital competency and well-being data were collected via a self-reported survey before and after the intervention.

**Results:**

Among the 4485 residents who were offered training, 67 (1.49%) expressed interest, of whom 12 (18%) of the 67 completed the training. The findings indicate a demand for basic digital training among social housing residents. The key findings revolve around the substantial dropout rate among those who were interested in undertaking the training. Barriers were strongly influenced by socioeconomic and health circumstances, reflecting the sociodigital inequalities commonly found in this group. For the training participants, the intervention was acceptable and achieved its goals, demonstrating the potential of tailored, persistent training efforts in overcoming barriers. There were no changes in self-reported well-being or digital competency outcomes (but this was limited by the small sample size).

**Conclusions:**

Sociodigital inequalities impact the reach, implementation, and acceptability of telephone-based digital training for social housing residents. Barriers to reaching and training digitally excluded groups can be overcome through the use of trusted intermediaries, personalized recruitment approaches, the minimization of administrative barriers, and tailored and agile training programs. Recognizing the resource-intensive nature of such initiatives, this study calls for enhanced recognition of intermediary efforts in national digital inclusion policies.

## Introduction

### Background

Facilitated by near-ubiquitous digital connectivity in the global north, digital technologies are increasingly used across all areas of life and bestow significant benefits upon those who use them [1,2]. In response, many organizations, not least public service providers, proceed as if access to the internet and digital technology is already universal [3]. However, although connectivity has increased, access to digital technologies and the competencies to use them are not yet universal [4,5]. In the United Kingdom, the 2021 Consumer Digital Index found that approximately 10 million people (14% of the population) do not have the basic digital competencies needed for use the use of everyday digital technologies [6]. Digital exclusion can no longer be seen as a product of rural or remote living, place-based infrastructural and connectivity issues, or older age. Instead, digital exclusion is currently most likely a product of wider social and economic inequalities. Sociodigital inequalities [7] refer to the interplay between traditional (ie, social, economic, and health) and digital inequalities and have led to systematic differences in the ability and opportunity of different groups to beneficially use digital technology and participate in society. These differences, in turn, contribute to deeper social inequalities, which lead to greater digital inequalities over time [8].

A growing body of literature has begun to document the extent of digital inequalities and highlight the specific barriers to digital use in different social contexts [8]. In the United Kingdom, the Digital Inclusion Strategy sets out how the government and partners from the public, private, and voluntary sectors will increase digital inclusion [9] by targeting the following 4 interconnected barriers to digital inclusion: digital access, skills, confidence, and motivation [10]. However, although national policies are imperative to improve inclusion from an infrastructure and digital connectivity [1] perspective, localized interventions are much better placed to address digital skills, confidence, and motivation across previously excluded groups [11]. In the United Kingdom, localized digital training interventions are typically delivered by intermediaries, such as voluntary and community sector organizations and public libraries, and include formalized group programs, one-to-one digital buddy programs [12], and peer support [13]. Specifically, the United Kingdom’s Local Government Association’s Digital Inclusion Programme is funding councils to reach residents and provide personalized digital training programs for those who do not have access to or confidence in using digital communication platforms [14]. However, there is limited evidence on whether these digital training interventions are effective in increasing “digital readiness,” the competency and increased motivation to use digital technologies [15-17]. However, evidence does suggest that individuals who participate in these digital interventions have a higher socioeconomic status, higher education, higher social participation, and a greater experience with technology [18,19]. Conversely, those who are already experiencing sociodigital inequalities remain the hardest to reach [20-22] owing to reported barriers, including physical health issues, a negative attitude toward technology, caring for a sick spouse, a lack of energy, and a lack of time [23,24].

### This Study

Within the context of the digital needs of hard-to-reach populations, this study focused on social housing residents in Southwest England. In the United Kingdom, social housing associations (HAs) are not-for-profit organizations that provide rental properties at 50% to 60% of market rates to those whose circumstances exclude them from the private market [25]. Overall, 3.9 million people live in social housing for various socioeconomic reasons [25]. Studies have shown that the demographic and socioeconomic profile of social housing residents means that they are significantly more likely to be digitally excluded and harder to reach than other groups in the United Kingdom [10,26-28]. This is because the factors that are known to increase digital exclusion are found at higher incidences among social housing residents than among those outside the social housing system [29,30]. These factors include lower incomes, fewer qualifications, older age, physical and mental health issues, disabilities, and living in more deprived areas [31,32].

The Smartline project [33] worked with 200 social housing households in one of the most deprived areas of England [34] to understand the potential of everyday digital technology to address health and well-being challenges. A qualitative scoping study on the feasibility and acceptability of digital technology among Smartline participants found that although the participants had positive perceptions of technology and were keen to try new technologies, digital readiness and the desired digital destination (goals) varied greatly among the community [28]. Several concerns surrounding technology use were identified, including data security and privacy concerns and the fear of “making a mistake” or “pressing the wrong button.” Many participants expressed a strong desire for further training and support.

Following this research, a training intervention was conducted to help Smartline participants get on the web and use digital communication technologies, such as web-based video calls and messaging, with confidence. The Getting Online: Staying Connected (GO:SC) intervention was originally planned as a face-to-face intervention with peer-to-peer support on how to use video calling technology. However, as with many research interventions during this time [35], the outbreak of the COVID-19 pandemic in March 2020 meant that the study had to be redesigned as a smaller-scale telephone-based training intervention for social housing residents delivered in conjunction with the Cornwall Council’s Digital Inclusion Team (DIT). The aim of this study was to evaluate the feasibility of the telephone-based training intervention. Specifically, informed by the feasibility framework of Bowen et al [36] and RE-AIM (Reach, Effectiveness, Adoption, Implementation, and Maintenance) model of Glasgow et al [37], this study aimed to evaluate the (1) reach and demand, (2) implementation, (3) acceptability, and (4) potential efficacy of the telephone-based digital training intervention for social housing residents [36,37]. These are established frameworks for evaluating digital health interventions [38,39] and are suitable and credible for testing an unexamined intervention in a real-life setting where constraints exist over conditions [36,38,39].

## Methods

### Overview of the Study Design

**Figure 1:**
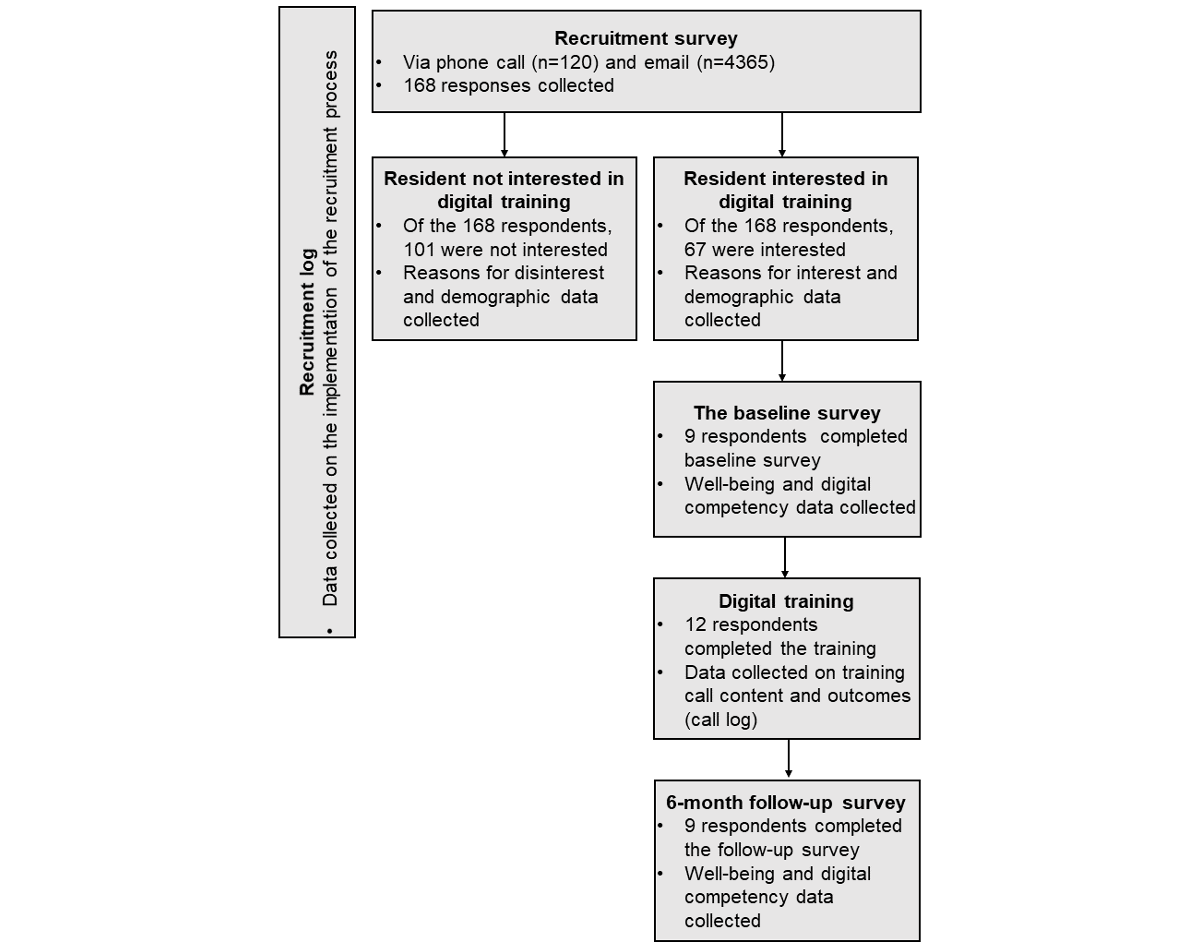
Feasibility study process map for the digital training program covering recruitment, data collection, and intervention delivery.

This was a mixed methods feasibility study. The protocol and ethics application were informed by best practices for the process evaluation of public health interventions [40]. An overview of the study procedure, including recruitment and data collection, is provided in Figure 1 and Table 1 and detailed throughout the subsequent subsections.

**Table 1:** Feasibility study data sources and time points of data capture.

Data source	Data captured	Time point of data capture	People who captured the data
Recruitment log	Participant ID Contact details, including the best time to contact and preferred method of contact Whether the participant has an internet-enabled device The date of completion of consent forms and surveys (administered at recruitment, baseline, and follow-up) Field notes on the recruitment process and intervention implementation	At recruitment	The research team
Recruitment survey (Multimedia Appendix 1; n=168)	Demographics (age, gender, disability, racial identity, and cultural identity) Interest in taking part (yes or no); reasons for taking part or not taking part Digital training interests (free text)	At recruitment	Participant
Well-being and digital competency survey (Multimedia Appendix 2; n=9)	General, emotional, behavioral, and social well-being Attitudes, behaviors, and competence regarding technology, including specific questions on video calls and messaging, and general technology questions	At baseline and 6 months after the intervention	Participant
Digital training call log (n=12)	The number of calls made Participants’ learning objectives The focus and content of training Outcomes Any other relevant notes on training	During and immediately after the training call	DIT^a^

^a^DIT: Digital Inclusion Team.

### Recruitment of Participants

Recruitment took place between October 2020 and June 2021. As we did not expect potential participants to have email addresses, recruitment was initially planned to take place via telephone, targeting only the Smartline participants. Participants who identified as having low digital competency or confidence in the Smartline baseline survey [41] were contacted via telephone and completed the recruitment survey asking respondents if they were interested in undertaking the digital training (Figure 1). The Smartline survey data enabled the analysis of the characteristics of those who did not respond and were not interested in the training offer.

As recruitment was lower than expected, it was decided to extend the intervention to all Coastline Housing residents in the greater Camborne area. Extending to all the residents of the HA meant that individual phone calls were no longer possible. The recruitment survey was sent to all Coastline residents who had an email address. A specially designed advertisement for digital training was also disseminated to the residents using the communication channels of the HA, including the residents’ monthly newsletter (printed and electronic), the HA’s Facebook (Meta Platforms, Inc) page, and printed posters placed in community areas. The recruitment survey asked the participants to outline their reasons as to why they were interested or not interested in receiving the training. The survey thus enabled the analysis of the characteristics of both the participants who were interested and participants who were not interested in the training intervention.

Residents who expressed an interest in the digital training either via telephone or email were posted the study information sheet, the consent form, and a baseline survey assessing well-being and digital competency (Multimedia Appendices 2-4). Participants returned the completed forms via a Freepost envelope to the research team, who forwarded the participants’ phone numbers and digital training interests to the Cornwall Council’s DIT via email. The DIT then directly telephoned each participant to arrange the provision of the training.

The inclusion criteria were adult (aged >18 y) social housing residents in Cornwall who defined themselves as lacking either the competency or confidence to use digital communication apps. These included existing participants in the Smartline project and residents of the wider Coastline HA. Participants were excluded if they did not have access to an internet connection or at least 1 internet-enabled device. Training was provided free of charge to participants (in line with the DIT’s protocol).

### The Intervention

The intervention is described in line with the TIDieR (Template for Intervention Description and Replication) standards for intervention reporting [42]. The training intervention was based on a 4-week, face-to-face, and basic digital skills course that was established by the DIT and delivered in libraries and community venues across Cornwall before the COVID-19 pandemic. The course structure adhered to the UK government’s Essential Digital Skills Framework [43], encompassing fundamental computer skills, problem-solving skills, communication skills, transactional skills, and skills for handling digital information and content, as detailed in Multimedia Appendix 5.

In response to the social distancing measures in place during the COVID-19 pandemic, the DIT adapted the training to be delivered over telephone. This adaptation was informed by best practices in digital inclusion initiatives [44] and participant preferences identified in our prior qualitative scoping work [28]. The adapted training approach emphasized informality; flexibility; person-centeredness; and task-specific, on-demand delivery, diverging from traditional, predetermined, and technically focused programs imposed by training organizations [44]. This informal model aligns with research indicating that training success factors encompass participant autonomy, personalized learning, practice opportunities, and individualized support [13,45]. Informal learning, in particular, enhances self-efficacy and proves highly effective for digitally marginalized groups [46].

The overall purpose of the training intervention was to support participants in achieving active and continued use of digital devices and communication apps of their choice (eg, WhatsApp [Meta Platforms, Inc], Zoom [Zoom Video Communications, Inc], and Facebook). The training was delivered through one-on-one phone calls facilitated by a digital inclusion officer from the DIT, all of whom possessed a background in education. To provide course content guidance and ensure consistent and best-practice training delivery, the DIT used a bank of task-specific instructions (ie, step-by-step guide to installing WhatsApp), which could be printed and posted to participants on demand (Multimedia Appendix 6). Training sessions varied in duration (between 5 min and 2.5 h) according to each participant’s individual learning needs, often including follow-up calls as necessary.

### Ethical Considerations

Overarching ethics approval to conduct research with Coastline Housing (a social HA in Cornwall) residents was granted by the University of Exeter Business School Research Ethics Committee as part of the Smartline project (eUEBS002996 v4.0). Specific approval for the GO:SC project was granted by the College of Life and Environmental Science Penryn Research Ethics Committee (eCORN002229). Written informed consent was obtained from all participants involved in the study (Multimedia Appendices 3 and 4). Data were anonymized using pseudonyms. Participants who completed the follow-up survey received a £10 (US $12.7) shopping voucher as compensation for their time.

### Data Overview

The following 4 qualitative and quantitative data sources were used to assess the feasibility and acceptability of the intervention: a recruitment log, recruitment survey, well-being and digital competency survey (administered at baseline and 6-month follow-up), and digital training call log (Table 1). Previously piloted by the Smartline participants, the well-being and digital competency survey (Multimedia Appendix 2) used quantitative rating scales and was completed at baseline (immediately before training commencement) and 6 months following the training, with the aim of assessing potential outcomes. This survey (disseminated via post) was based on a validated survey, the “Happiness Pulse” [47], and included 4 domains of psychological well-being (general, emotional, behavioral, and social). A bespoke module on digital attitudes, behaviors, and competence was developed by the research team for the wider Smartline project. This digital module was informed by behavior change and technology acceptance theories [48-51] and included questions adapted from existing sources, including the UK government’s Digital Inclusion Evaluation Toolkit [52]. The module contained specific questions on video calls and messaging in addition to questions on technology in general. The theoretical basis of this module is provided in Multimedia Appendix 7 [47,49-63].

### Data Analysis

To determine the reach of and demand for the intervention, as well as participants’ levels of engagement with the training, descriptive statistics (frequencies and percentages) from the recruitment log, recruitment survey (n=168), and training call log (n=12) were calculated. A probit model was used with the recruitment survey data to identify the socioeconomic factors associated with initial interest in participating in the digital training program. The analysis was performed in Stata (version 17; StataCorp) [64].

For the well-being and digital competency survey, the scoring protocol for the Happiness Pulse was followed [53], with means and SDs calculated from summary scores to describe each of the 4 well-being domains. As digital competency outcomes were measured on interval scales, medians and IQRs were calculated for these outcomes.

The COREQ (Consolidated Criteria for Reporting Qualitative Research) criteria [65] for reporting qualitative research were adhered to throughout the analyses. Qualitative data analysis used data from the recruitment log, recruitment survey, and digital training call log. To manage the qualitative data analysis process with transparency and traceability [66], NVivo (QSR International) [67] was used. In line with best practices [68], 3 rounds of inductive coding were conducted using a constant comparative method [69,70]. The first round of coding was open and focused on identifying and labeling discrete incidents. For example, “I have a smartphone, but I struggle to use it” contains 2 incidents: an object (smartphone) and a construct (competency). The second round of coding was axial, where open codes were compared (via contradiction, expansion, and support) and integrated into themes, and the third round of coding was selective, where connections between themes were compared and refined to build the grounded theory. The lead researcher (TW) conducted the initial coding, and the themes identified were discussed with a second researcher (SAB). To further improve rigor and reliability [71,72], a third researcher (KM) was consulted, and minor discrepancies were resolved through discussion. Pseudonyms were applied to protect participants’ identities.

The quantitative and qualitative data were integrated within the selected feasibility criteria of reach, demand, acceptability, implementation, and potential outcomes [36] to provide a complete picture of the feasibility, acceptability, and potential impact of the intervention. This use of mixed methods enabled triangulation to strengthen the validity of the findings and complementarity to explore different facets of a phenomenon [73].

## Results

### Reach and Demand of the GO:SC Digital Training Intervention

#### Recruitment and Reach

Digitally excluded groups within social housing are known to be difficult to reach [20,26]. In total, 4485 social housing residents were offered the training either via email (n=4365, 97.32%) or phone call (n=120, 2.68%; Table 2). The total number of responses to the recruitment survey (conducted via phone and email) was 168. A much higher proportion of phone survey respondents (37/120, 30.8%) were interested in the training, compared with the proportion of email survey respondents who were interested (30/4365, 0.69%). Although the HA actively promoted the intervention via its various web-based and printed communication channels over several months, none of the residents responded to these advertisements.

**Table 2:** Demand for and uptake of the digital training in and demographic characteristics of each group^a^.

	Total (n=168), n (%)	Women residents, n (%)	Men residents, n (%)	Residents with missing gender data, n (%)	Residents aged 18 to 24 y, n (%)	Residents aged 25 to 34 y, n (%)	Residents aged 35 to 44 y, n (%)	Residents aged 45 to 54 y, n (%)	Residents aged 55 to 64 y, n (%)	Residents aged 65 to 74 y, n (%)	Residents aged ≥75 y, n (%)	Residents with missing age data, n (%)
Not interested in the training	101 (60.1)	55 (54.5)	35 (34.7)	11 (10.9)	4 (4)	10 (9.9)	12 (11.9)	14 (13.9)	15 (14.9)	22 (21.8)	7 (6.9)	17 (16.8)
Interested in the training	67 (39.9)	41 (61.2)	23 (34.3)	3 (4.5)	0 (0)	3 (4.5)	5 (7.5)	8 (11.9)	17 (25.4)	24 (35.8)	8 (11.9)	2 (3)
Interested in the training but did not complete the training	55 (82.1^b^)	31 (56.4)	21 (38.2)	3 (5.5)	0 (0)	3 (5.5)	5 (9.1)	8 (14.5)	12 (21.8)	19 (34.5)	6 (10.9)	2 (3.6)
Interested in the training and completed the training	12 (17.9^b^)	9 (75)	3 (25)	0 (0)	0 (0)	0 (0)	0 (0)	0 (0)	5 (41.7)	5 (41.7)	2 (16.7)	0 (0)

^a^The percentage values in columns 3 to 13 were calculated using the corresponding n value in column 2.

^b^The percentage value was calculated with 67 as the denominator.

Phone calls were the most successful means for recruiting older and more digitally excluded groups. This success can be attributed to their familiarity as a channel for communication and the person-centered approach they enable [44]. Phone conversations also facilitated a further understanding of personal circumstances (eg, social, financial, or health needs) and desired outcomes (eg, connecting with friends or family or accessing health services), making it easier to adapt the conversations to include relevant training benefits. Furthermore, a previous study [74] found that women tend to be more receptive to recruitment contact via phone, which may explain the gender bias found.

Overall, 39.9% (67/168) of the recruitment survey respondents were interested in potentially undertaking the intervention. Among those initially contacted via email and phone, older women residents and those contacted by the HA were more interested in the intervention (probit model; Table 3). None of the interested participants who responded to the email survey completed the training. Our results affirm the substantial challenge of reaching and recruiting individuals interested in foundational digital training using conventional communication channels.

**Table 3:** Probit model analysis results, that is, factors associated with the initial interest in the digital training intervention (n=168)^a^.

Explanatory variable	Description of variables	Coefficient	*P* value
Contacted by HA^b^ (dummy)	If contacted by HA	0.863	.001
Disable (dummy)	Respondent is disabled=1	0.228	.55
Age55+ (dummy)	Age > 55 years	0.828	.003
Disable Age55+	—^c^	0.221	.66
Women (dummy)	Respondent is women=1	0.105	.63
Cornish (dummy)	Respondent’s ethnic group is Cornish=1	0.009	.98
English (dummy)	Respondent’s ethnic group is English=1	0.135	.72
British (dummy)	Respondent’s ethnic group is British=1	0.159	.64
Constant	—	−1.314	<.001
Log Likelihood	—	−101.080	—
Number of respondents	—	168	—

^a^Dependent dummy variable: interested in participating.

^b^HA: housing association.

^c^Not available.

#### Digital Training and Reach

Of the 37 potential participants who indicated interest via phone, 12 (32%) completed the training. Of these 12 participants, 9 (75%) were women. Only respondents aged ≥55 years undertook the training, of whom 42% (5/12) were aged 55 to 64 years, 42% (5/12) were aged 65 to 74 years, and 17% (2/12) were aged ≥75 years (Table 2). Overall, 50% (6/12) of the participants reported a disability, 42% (5/12) of the participants reported no disability, and 8% (1/12) of the participants were missing data on disability. The majority of the participants racially identified as White (9/12, 75%) and culturally identified as British (5/12, 42%) or British Cornish (4/12, 33%). The demographic, disability, racial identity, and cultural identity profiles of participants who completed the training were proportionally similar to those who did not complete the training.

The small sample size recruited for digital training is consistent with the small sample sizes recruited to other feasibility studies on interventions for digitally excluded populations. For example, Barbosa Neves et al [39] recruited only 12 participants in residential care to a feasibility study concerning the use of digital communication for social connectedness. Nonetheless, given their research objectives focused on uncovering feasibility, this sample size was appropriate and useful [36,75]. Similarly, we argue that our findings are useful for interpreting the feasibility of this digital intervention among social housing residents.

#### Training Demand

The most salient factors influencing the demand for the intervention were digital competency, preference for nondigital communication, and social networks. These multifaceted factors underscore the intricate dynamics surrounding digital training interventions and highlight the need for tailored strategies to address diverse participant needs and circumstances.

Among those interested in undertaking the intervention, demand was highest for training on video calling, primarily using Zoom; setting up and using devices, primarily a tablet; and improving digital skills, knowledge, and confidence in general, with most participants (37/67, 55%) who indicated interest in the recruitment survey noting multiple training needs across the 3 areas. In line with the initial scoping study conducted by Buckingham et al [28], it was a lack of competency in using digital devices, rather than device ownership and internet connectivity, that hindered digital technology use: “I am not able to use it [device] properly” (James, a man resident aged 55 to 64 years) and “I am interested in video calling, I have a smartphone, laptop, tablet, and mini-iPad, but I struggle to connect to the internet” (Mary, a woman resident aged 55 to 64 years).

The primary reason people were not interested in accessing the training was because they were already competent in using web-based video calling and messaging tools (57/101, 56.4%). However, those who were already competent were supportive of the intervention, reporting a need for digital training in general. Further reasons for the lack of demand included preferences for nondigital communication technology (“I prefer to just pick the phone up and call people” [Thomas, a man resident of unknown age]) and a feeling that the training was not personally necessary (“No, don’t think I need to learn things like this at my age, manage just fine thanks” [Deborah, a woman resident aged 65 to 74 years]).

Social networks played a key role in demand among participants who replied to the initial recruitment survey about the intervention. The lack of a digitally engaged social network was commonplace among those contacted, as was a small social network in general: “I don’t know anyone who I would call. I only have my sister and she doesn’t use internet stuff” (John, a man resident aged 55 to 64 years). Unsurprisingly, living farther away from family and friends was a key reason for engaging with digital communication technology: “My family live a distance away, so keeping in touch is important” (Sharon, a woman resident aged 86 years). However, for us, a key finding was that people remain happy to rely on their broader support group, particularly younger family members. Having family and friends that could be relied on for help was a disincentive to undertake the training: “No help necessary, grandchildren able to help” (Rebecca, a woman resident aged 65 to 74 years).

Regarding the overall reach of the intervention, although initial recruitment calls found a moderate level of demand for this training, this interest did not translate into the same level of reach, with only 18% (12/67) of the interested participants completing the training. A key reported factor contributing to the lack of reach was health status, either personal health status (12/67, 18%) or the health status of family members (9/67, 13%); however, long-term disability (23/67, 34%) or impairments also affected the capacity to use technology and communicate, particularly among participants with visual, speech, or hearing difficulties (4/67, 6%). Caring responsibilities also acted as an impediment to uptake, particularly among older women:

I am interested [in the training], but my head is just full of decisions about my husband who will be in a nursing home for the rest of his life, I need to focus on what is important to me now.

Martha, a woman resident aged 55 to 64 years

### Implementation of the GO:SC Digital Training Intervention

The main implementation finding relates to the reduction in the number of participants between those who expressed initial interest in the training and those who completed the training. The study found that key facilitators included the HA’s established relationship with a digitally susceptible population and the flexible, informal approach of the DIT. Barriers involved issues with written consent, internet access, and device functionality. Here, we discuss each of these in turn.

The tangible and intangible positive roles that the HA played as an intermediary [76,77] in the implementation of the intervention cannot be overstated. In the United Kingdom, the role of HAs has evolved over recent decades to include supporting the health and well-being of their residents [78]. As a result, many HAs have built meaningful, trust-based relationships with their residents, and this factor played an important role in this project. From a practical perspective, this meant that the collaboration of Smartline with the HA enabled a wider reach in advertising the training offer. For example, the HA was able to use its customer relationship management system with phone numbers and email addresses to contact over 4000 residents. Indeed, examining the socioeconomic factors associated with initial interest in participating in the digital training program revealed that respondents who were contacted by the HA were more likely to be interested in the training program (*P*<.001; Table 3).

Another factor important for reach was customer liaisons by the HA with known susceptible residents who they felt would be interested in and would benefit from the training. Although recruitment remained difficult and the uptake of the intervention was low, the implementation of the recruitment survey and associated data collection would not have been possible without the help of the HA.

Participant dropout at the recruitment stage negatively affected implementation; only a small proportion of those who expressed an initial interest went on to complete the training. Among those (n=67) who were initially interested in the training, many (n=37, 55%) required multiple callbacks (up to 4) before they could be reached again; for reasons discussed next, many (55/67, 82%) dropped out. Making multiple callbacks was an administratively complex task that required many hours and email exchanges within the recruitment team. The need for written consent was a key factor for recruitment dropout, as was the need for a working digital device and an internet connection. Completing and returning the necessary consent forms was found to be a particular challenge for some participants. Participants noted that “filling in forms is a worry” (Julia, a woman resident aged 65 to 74 years) and that making time for the task was difficult: “I have the forms but have a lot on at the moment” (Jess, a woman resident aged >75 years). The possession of an internet-enabled device and internet connection was a requirement for participation in the training. Although we found high rates of digital technology ownership and internet connection possession among the participants contacted, 17.3% (29/168) of the respondents to the recruitment survey did not have a tablet, 5.9% (10/168) of the residents did not have a smartphone, and 7.1% (12/168) of the residents did not have an internet connection. Financial constraints, in particular, were a key reason for the lack of internet:

If I could get internet I would be interested [in the training], but not at the moment due to affordability issues.

Thomas, a man resident of unknown age

I live in poverty so am scared I will incur charges using the tablet and smartphone.

James, a man resident aged 55 to 64 years

For other interested participants, the working condition of the device was also a barrier to participation in the training:

I need to learn how to adjust settings so my old PC can cope.

David, a man resident aged 55 to 64 years

I am not sure if my phone is smart enough.

Grace, a woman resident aged 55 to 64 years

In addition, it is important to reflect on the implementation of the training itself. In practice, 3 different digital inclusion officers delivered the telephone-based training to 12 participants. The calls lasted between 5 minutes and 2.5 hours. To fit around participants’ caring responsibilities and day-to-day lives, all participants required multiple calls to arrange and rearrange training times. Training sessions lasted as long as required for the participant to learn to use the digital application, and follow-up support calls (up to 5) with the digital inclusion officer were arranged to ensure that the training objectives were achieved (Table 4). Successful delivery of the training required flexibility and persistence from the DIT. This indicates that there is a wide range of digital needs that are best served by informal and one-to-one support directed by individual needs [13,46].

**Table 4:** Overview of participants’ training objectives and associated outcomes.

Participant	Training objectives	Digital training needs	DIT training calls, n	Outputs	Outcomes
Mary, a woman resident aged55 to 64 years who reported having a disability	To be able to video call daughters	Needed help connecting a device to the internet and to learn how to video call	1	Received training to connect device to the internet and video calling	Uses video calls to talk to her daughters at dinnertime every week
Julia, a woman resident aged 65 to 74 years who reported having a disability	To order medicine for disabled son, send flowers to family members, and get inspiration for arts and craft projects	Needed to learn how to use emails, use web-based prescriptions, make web-based purchases, perform an internet search, and browse a website	5	Received training on web-based form-filling and purchasing, and internet searching.	Ordered flowers for family members and ordered medication for her son
Jade, a woman resident aged 55 to 64 years who reported having a disability	To be able to video call	Needed to learn how to unmute the computer microphone and how to video call	1	Received video call training	Increased competence in video calling
Susan, a woman resident aged 55 to 64 years who reported having a disability	To be safe and secure on the web, digitally record and mix music, and improve digital competency	Needed to learn about web-based safety and security and about different music recording software products, help purchasing music recording software, help setting up music recording software, and help connecting guitar mic to the computer and recording software	4	Received training to purchase music recording software, set up the music recording software, and connected guitar mic to the computer and audio recording software	Increased competence in recording and mixing guitar playing audio
Grace, a woman resident aged 55 to 64 years	To be able to video call daughter	Needed to purchase a device through which a video call could be made, help sorting passwords to activate internet connection, help connecting a device to the internet, and to learn how to video call	1	Received training to fix password issue, activate internet connection and video calling	Video calls her daughter
Rosie, a woman resident aged 65 to 74 years	To record and mix audio from a singing group	Needed to learn about different music recording software products and learn how to use software to merge several singing voices together	1	Received training on using software to mix multiple audio tracks	Mixes tracks together for a barbershop singing group
Tracy, a woman resident aged 55 to 64 years	To be digitally competent	Needed help completing the Learn My Way digital training course	3	Supported to complete the Learn My Way digital training course	Increased digital competence
Jess, a woman resident aged >75 years	To better manage digital communication administration for a church group	Needed a refresher on using Zoom, to learn how to attach pictures to emails and save emails to folders, and to learn how to rearrange text and line gaps in Microsoft Word	3	Received training on attaching pictures to emails, saving emails to folders, and using Microsoft Word	Increased ability to manage digital communication administration for the church group
Daniel, a man resident aged 65-74 years	To be able to video call mother and print envelopes	Needed help setting up video call software and guidance on printing	1	Received video call training and guidance on printing	Video calls to mother and prints envelopes
Paul, a man resident aged 65 to 74 years	To be able to video call family and friends	Needed help setting up video call software	1	Received video call training	Ability to video call
Holly, a woman aged >75 years	To be able to keep in touch with family	Needed help connecting a device to the internet, help sorting intermittent internet problems, help navigating device settings to complete software update, and to learn how to video call	2	Received training on connect a device to the internet and sort intermittent internet problems	Unresolved internet connection issue
Mark, a man resident aged 65 to 74 years	To find out about events and news in local area, keep in touch with family and friends, use the Coastline Housing app to report maintenance issues with the property	Needed to learn how to use social media to connect with local news and event groups and to keep in touch with family and friends, how to video call, and how to install and set up apps	2	Received training on how to use social media, fix several problems with devices, installation and setup of apps, accessing and using a Gmail account, purchase of a new device for video calling, and video calling	Increased digital competency, video calling family, and using the Coastline Housing app to report issues with the property

### Acceptability of the GO:SC Digital Training Intervention

Similar to the key findings on implementation, the study’s key findings on acceptability revolve around the substantial dropout rate, highlighting the challenges of translating training interest to training participation. Participants dropped out because of competing priorities, including health issues, caregiving responsibilities, and time constraints. However, all the 12 participants who started the training completed it; this suggests high acceptability of the intervention itself.

As with all research conducted during this period, the COVID-19 pandemic impacted all aspects of the intervention. We found the lockdown to have both a positive and negative impact on acceptability. For some, it was a driver for learning how to use video calls to access health services, social groups and classes, and resident groups, which had transitioned to function on the web:

I need help to connect to video appointments with the health professionals helping me.

Katy, a woman resident aged 25 to 34 years

I would really like to join the online Coastline meetings [HA residents’ group] but don’t know how to use Zoom. It’s a priority for me to get online now.

Lilly, a woman resident aged >75 years

The lockdown also had a negative impact on the acceptability of the training intervention, with potential participants noting a need to attend to everyday tasks and self-care, rather than learning new skills: “I have been unwell with COVID for months, I just need to focus on the day-to-day things at the moment” (Judith, a woman resident aged 45 to 54 years). Importantly, we found that the pandemic compounded and increased several preexisting barriers to undertaking digital training for our participants. For example, we found that preexisting health conditions arose as a key barrier to participation:

I’m having a few health issues at the moment, call back in a few months.

Linda, woman resident aged 65 to 74 years

Participant’s lives were generally “busy” (Diana, a woman resident aged 65 to 74 years), and they did “not have time for this at the moment” (Ruth, a woman resident aged 55 to 64 years) or needed to focus on caring responsibilities. Regarding the acceptability of the training vis-à-vis the everyday lives of our participants, 2 further themes emerged: the method used to deliver the intervention and the timetabling of the training sessions. Understanding that the Smartline participants had a strong preference for peer-based, face-to-face activities [27], participants’ concerns about the effectiveness of support being delivered over the phone were expected:

I am apprehensive about if a phone conversation would be enough to get online, would prefer one-to-one and face-to-face. I learn best by doing.

Michael, a man resident aged 65 to 74 years

I need baby steps with the learning as I am not confident with technology, AKA a technophobe.

Gill, a woman resident aged 65 to 74 years

The timing of the training was an important factor for participants, particularly for those who were working or had chronic health issues: “I have ME [myalgic encephalomyelitis or chronic fatigue], so afternoon is better for me” (Susan, a woman resident aged 55 to 64 years). Therefore, despite the increased need for digital communication at this time [79], the already complex social and health needs of the Smartline participants [80] meant that the recruitment and retention of interested participants was time intensive and a major challenge for the acceptability of the intervention.

### Outcomes of the GO:SC Digital Training Intervention

Of the 12 people who participated in the training, 9 (75%) completed the baseline and follow-up well-being and digital competency surveys (Multimedia Appendix 2). Table 5 provides the summary well-being scores for participants who undertook the digital training intervention; there were no changes in the mean general, emotional, or social well-being between baseline and follow-up.

Counter to the overall aim of the intervention, we found a small reduction in behavioral well-being for participants who had undertaken the intervention [53]. The behavioral well-being measures included a question asking whether participants were attending courses and a further question on whether respondents were learning a new skill. As such, respondents would have indicated “yes” to these questions at baseline; however, at follow-up, participants were unlikely to be undergoing any other training given the ongoing COVID-19 restrictions. As such, this decrease could be explained by a confounding reduction in training levels from before to after the intervention, rather than an actual decrease in behavioral well-being.

Table 6 provides the summary digital competency scores of the intervention participants (n=9) at baseline and follow-up. From Table 6, it can be inferred that there were no clear changes over time in any of these scores.

**Table 5:** Summary baseline and follow-up well-being scores for participants who undertook the digital training intervention (n=9)^a^.

Outcome	Intervention, mean (SD)	
	Baseline	Follow-up
General well-being	5.44 (2.36)	5.50 (2.51)
Emotional well-being	6.43 (1.78)	6.38 (1.96)
Behavioral well-being	6.85 (1.83)	5.70 (2.26)
Social well-being	6.75 (3.68)	6.78 (3.85)

^a^Higher scores indicate higher well-being in each domain. The range was 0 to 10 for all domains.

**Table 6:** Summary digital competency scores for participants who undertook the digital training intervention (n=9).

Digital module question^a^			Intervention, median (IQR)		
			Baseline		Follow-up
**Video calling and messaging questions**					
	Frequency of use^b,c^	5 (4-6)		5 (2-6)	
	Perceived ease of use	3 (3-4)		3 (3-4)	
	Perceived usefulness	4 (3-4)		3 (3-4)	
	Perceived reliability	3 (3-4)		3 (3-4)	
	Intentions to use	4 (4-4)		4 (3-5)	
	Autonomy	2 (2-4)		3 (3-3)	
	Feeling close to others	4 (2-4)		3 (3-4)	
	Desire to use technology, as friends are using it	3 (2-4)		3 (3-4)	
	Desire to use technology, as family is using it	4 (4-4)		4 (3-4)	
	Other people think that I should use it	4 (3-4)		3 (3-4)	
**General technology questions**					
	General technology self-efficacy	4 (2-4)		3 (2-3)	
	Enjoyment of using technology	3 (2-4)		3 (2-3)	
	Self-rated ability to use the internet^b^	3 (3-3)		4 (3-4)^d^	
	Perception that the internet makes life easier	3 (3-4)		4 (3-4)	
	Self-rated ability to use smartphones^b^	3 (3-3)^e^		3 (3-4)^f^	
	Frequency of searching online for health information^b,c^	2 (1-2)		2 (1-2)	
	There are people I can talk to online if feeling lonely	2 (2-4)		2 (1-3)	

^a^Higher scores indicate higher perceived competence, greater frequency of use, and more positive attitudes toward technology.

^b^Reverse-coded responses were used for these questions.

^c^Scores range from 1 to 6 for the frequency of use questions; scores range from 1 to 5 for all other questions.

^d^n=7 (data are missing; therefore, the number of responses is indicated).

^e^n=6 (data are missing; therefore, the number of responses is indicated).

^f^n=5 (data are missing; therefore, the number of responses is indicated).

Although conclusions on potential efficacy based on the survey data are limited owing to the small sample size, the qualitative data indicated that participants had achieved their original training objectives. Table 4 provides a summary of the training objectives, training needs, and the level to which they were met.

From Table 4, it can be inferred that increased competency in using video calling apps, particularly help with the installation and setting up of these apps, were key training needs. Participants’ training needs were motivated by both social and personal goals, such as contacting family members and becoming more competent with digital technology in general. Although advertised as training on video calling and messaging, participants received a diverse range of training, from training on how to order prescriptions on the web to training on more complex tasks such as recording and mixing music using web-based platforms. The flexibility to deliver such diverse and tailored training was not initially planned as part of the intervention yet proved a successful strategy. Overall, from the qualitative data, we found that participants achieved their training objectives and social and personal goals and have the potential to use other digital technologies in the future.

## Discussion

### Principal Findings

The findings indicate a demand for basic digital training among social housing residents, and the intervention was acceptable for those who received it. However, recruitment and implementation were challenging, with potential participants experiencing barriers that reflected the sociodigital inequalities commonly found in this group [10,26-28]. Barriers were strongly influenced by socioeconomic and health circumstances, which were closely related to preexisting digital readiness (eg, preexisting skills, confidence, motivation, and access) and further compounded by the COVID-19 pandemic. Our results confirm that the factors known to increase digital exclusion are particularly high among social housing populations [29,30] and highlight the interplay between traditional inequalities (ie, social, economic, and health) and digital inequalities [7]. However, social and personal goals were achieved by the participants who received the intervention. This demonstrates that tailored, flexible, and persistent training efforts can overcome barriers.

### Implications for Policy and Recommendations for Practice

Regarding policy, the UK’s Digital Inclusion Strategy aims to “equip the whole country with the skills, motivation and trust to go on the internet, be digitally capable and to make the most of the internet” [81].

Although national policies are imperative to improve infrastructure, access, and digital connectivity [1,9,10], the implications of this study are that an effective policy also needs to focus on strategies for reaching digitally excluded groups [20-22]. The essential strategies and recommendations for practice derived from our findings are listed in Textbox 1.

**Essential strategies and recommendations for practice.**

1. Partnerships with trusted intermediaries: forge partnerships with trusted local intermediaries, such as housing associations (HAs), community organizations, councils, and public libraries [76,77]. Prioritize intermediaries with established relationships and direct contact with the target group for effective reach.
2. Personalized recruitment approaches: use personalized recruitment methods, such as personal phone calls or face-to-face conversations. Understand individuals’ social, economic, health, and digital circumstances and align training benefits with their specific goals [7].
3. Minimize administrative barriers: reduce administrative burdens by minimizing form-filling processes, which negatively impact recruitment efforts. Be mindful of research monitoring procedures that may affect recruitment numbers, aiming for a streamlined approach.
4. Tailored and agile training programs: offer a flexible combination of device provision and internet access tailored to individual needs. Implement agile, person-centered training programs that adapt to participants’ personal goals and requirements.
5. Resource allocation and recognition: recognize the resource-intensive nature of initiatives targeting digitally excluded groups. Advocate for a stronger recognition of the efforts and resources required by intermediaries in national digital inclusion policies.

By implementing these strategies, policy makers, organizations, and communities can address sociodigital inequalities. However, in making these recommendations, we recognize that this places a considerable burden on individuals delivering such interventions. Future feasibility research of this nature could investigate the burden on intervention deliverers and the associated economic costs, which were not examined here.

### Limitations

A strength of this study is its focus on social housing residents, an understudied population with associated socioeconomic inequalities that can present particular barriers to digital technology use. Another strength of this study is the collection of quantitative and qualitative data from various sources to enable rich insights into feasibility, acceptability, and potential impact, including the capture of data on those who initially expressed an interest in participating but did not go on to receive the intervention.

A limitation of the study is the small sample size for quantitative evaluation; however, as this is a feasibility study, quantitative outcomes (well-being and digital competency) were only intended to be indicative of the potential impact and were supplemented by qualitative findings on the achievement of training objectives. Owing to the unexpected difficulties in recruiting participants to the intervention, follow-up interviews were not possible within the time frame of the project.

We measured psychological constructs with individual items to balance theory alignment with reducing participant burden. However, we suggest that future feasibility and acceptability studies use established multiitem measures to assess such constructs. The final limitations to note are those with regard to intervention delivery fidelity and economic costs. The study did not assess how the DIT delivered the intervention, other than following the “standard operating procedure.” It is possible that variations occurred in training delivery with regard to relationships with participants. Future studies should consider structured approaches to assessing intervention fidelity [82].

Finally, the intervention’s cost could not be specified, as it was provided through a county council’s DIT. Despite their personalized nature, similar personalized digital training programs are common in UK councils [14]. Therefore, the results of this study are valuable for providers facing challenges in engaging specific resident groups.

### Conclusions

This study contributes to the contemporary literature, theory of “sociodigital inequalities” [7], and need to redefine digital inequalities in terms of their relation to other forms of socioeconomic and health inequalities. To address sociodigital inequalities, this study highlights that future policies need to be more proactive in reaching excluded groups, and such initiatives need to be considerate of people’s everyday lives, which will be conditioned by social and health circumstances. To achieve this, initiatives need to be appropriately resourced and include a flexible combination of digital provision with an agile person-centered approach to training based on personal needs and goals.

## Appendix

Multimedia Appendix 1 Recruitment survey.

Multimedia Appendix 2 Well-being and digital competency survey.

Multimedia Appendix 3 Information sheet.

Multimedia Appendix 4 Consent form.

Multimedia Appendix 5 Course content guidance.

Multimedia Appendix 6 Task-specific leaflet example.

Multimedia Appendix 7 Theoretical basis of digital module.

## Abbreviations

COREQ: Consolidated Criteria for Reporting Qualitative Research
DIT: Digital Inclusion Team
GO:SC: Getting Online: Staying Connected
HA: housing association
RE-AIM: Reach, Effectiveness, Adoption, Implementation, and Maintenance
TIDieR: Template for Intervention Description and Replication

## Data Availability

The anonymized quantitative data sets analyzed during this study are available from the University of Exeter Open Research Exeter repository. Qualitative data cannot be shared because they contain sensitive participant information, identifying information, and information whose dissemination would violate the agreement to which the participants consented.
