# Supplementary material for: Telephone-Based Training Intervention for Using Digital Communication Technologies for Social Housing Residents During the COVID-19 Pandemic: Mixed Methods Feasibility and Acceptability Evaluation
Source: JMIR Form Res. 2024 Jan 26;8:e45506. doi: 10.2196/45506 (PMC10858426; doi:10.2196/45506)
Supplement: Multimedia Appendix 1 [file formative_v8i1e45506_app1.docx]

**Recruitment survey introduction:**

Would you like help getting online, for example to use video calling or social media to connect with others, or know a friend or family member who might? We can help!

Smartline is running a project to help people get online and use video calling or messaging software with confidence; for example WhatsApp, Zoom, or Facebook messenger. The help is given via one-to-one phone calls experienced people at Cornwall Council. Anyone is welcome to join whether you are a complete beginner or someone wishing to expand your online skills.

If you don’t have a device and internet connection we can provide this for you. If you decide to take part you can receive a £10 shopping voucher.

If you are interested in digital support, or you know someone who would be, please complete the form (click the button below).

If you are not interested, we would really appreciate you completing the form to tell us why as this will help the research into how we support people with getting connected online.

[button: COMPLETE FORM]

With thanks from the Smartline team

Find out more: <https://www.smartline.org.uk/>

**Recruitment Survey**

- *Question piping to be used*

1. Are you interested in participating in Getting Online: Staying Connected for yourself or on behalf of someone else?

- Yes <pipe to 1a>
- No <pipe to 1b>

*If Yes >*

1a. What is the main thing you would you like support with?

For example setting up a device, using online video call services, or using online shopping services

*Text Box*

<pipe to 3>

*If No >*

1b. What is the main reason you are not interested?

- I am already as competent as I need to be using digital technology
- I have a family member or friend who helps me with digital technology
- I am not interested in using online messaging or video call services
- I have health issues or I am caring for someone with health issues
- I have other priorities in my life at the moment
- I do not have internet access or a device to access the internet <pipe to 2>
- Other (Open Text box)

<all other responses pipe to 5 demographics>

*Routed from “I do not have internet access or a device to access the internet”*

1. If we were able to supply you with an internet-enabled tablet would you be interested in joining the study?

- Yes <pipe to 1a>
- No <go to 4. optional demographics, thank you>

1. Contact info - if you are registering on behalf of someone else, please provide your own contact information initially

Name:

Address:

Postcode:

Email:

Telephone number:

1. It would be really helpful if you can spare the time to also complete this optional information. This helps us understand who we are communicating with. Your data is stored securely and is never shared outside the project. Demographic information is collected anonymously. You can skip this question if you prefer, but please press <submit> or your response will not be recorded.

- Gender (*tick box*) - Male, Female, Non-binary, Prefer not to say
- Age (*tick box*) - 18-24, 25-34, 35-44, 45-54, 55-64, 65-74, 75+, Prefer not to say
- How would you describe your racial identity? (*open text box*)
- How would you describe your national or cultural identity? (*open text box*)
- Do you consider yourself to have a disability? (*tick boxes*) - yes, no, prefer not to say

*Include a thank you screen after submit.*

Thank you for completing this survey! If you have any questions relating to the responses you have provided please contact the Smartline team on [smartlineresearch@exeter.ac.uk](mailto:smartlineresearch@exeter.ac.uk)
