# Supplementary material for: Telephone-Based Training Intervention for Using Digital Communication Technologies for Social Housing Residents During the COVID-19 Pandemic: Mixed Methods Feasibility and Acceptability Evaluation
Source: JMIR Form Res. 2024 Jan 26;8:e45506. doi: 10.2196/45506 (PMC10858426; doi:10.2196/45506)
Supplement: Multimedia Appendix 2 [file formative_v8i1e45506_app2.pdf]

Thank you for agreeing to complete this survey as part of the Smartline project. We are interested in finding out if and how your wellbeing and views on digital technology change over time. Your answers will be confidential and only available to the Smartline team. If you have any queries please contact the Smartline team on **smartlineresearch@exeter.ac.uk**

It is important to answer ALL of the questions. The survey will take approximately 20 minutes to complete in total.

The first part of the survey is The Happiness Pulse; a short survey which measures different aspects of wellbeing – "GENERAL WELLBEING", "BE", "DO" and "CONNECT".

In the second part of the survey you will be asked some additional questions about "DIGITAL TECHNOLOGY", including how you use digital technology and how you feel about it.

If you feel you are struggling more deeply with your mental health and wellbeing please call Samaritans on 116 123.

How will my information be handled?

The University of Exeter processes personal data for the purposes of carrying out research in the public interest. The University will endeavour to be transparent about its processing of your personal data and this information sheet should provide a clear explanation of this. If you do have any queries about the University's processing of your personal data that cannot be resolved by the research team, further information may be obtained from the University's Data Protection Officer by emailing dataprotection@exeter.ac.uk or at www.exeter.ac.uk/dataprotection

After completing the wellbeing and digital skills survey, your information will be anonymised and processed and coded with study numbers so no names will be retained. The anonymised and processed data will be stored in a repository and destroyed in 2033 as required by the funder. Only the research team will have access to a list containing identifiable information such as your name, address, phone number, study number, so we can contact you during the study if needed. This list will be securely stored on the University server with access limited to the core research team. This information will also be destroyed on completion of the larger Smartline project in December 2033 in accordance with University of Exeter's document retention polices.

Please be aware that you can withdraw from the study at any time. If you withdraw from the study, data collected up to the point of withdrawal may be retained and used by the project in an anonymized format.

If you have any concerns regarding the handling of your data or would like your consent withdrawn at any time, please contact the research team on smartlineresearch@exeter.ac.uk

Part 1: Happiness Pulse survey

For us to store your data in an anonymised format we need to create a unique identifier code. Please could you provide the following information.

First name

Middle name (if appropriate)

Last name

Date of birth

Date **Month** **Year**

SECTION 1: GENERAL WELLBEING

1. Overall, how satisfied are you with your life nowadays?

Not at all ☐ 0 ☐ 1 ☐ 2 ☐ 3 ☐ 4 ☐ 5 ☐ 6 ☐ 7 ☐ 8 ☐ 9 ☐ 10 Completely

2. Overall, to what extent do you feel the things you do in your life are worthwhile?

Not at all ☐ 0 ☐ 1 ☐ 2 ☐ 3 ☐ 4 ☐ 5 ☐ 6 ☐ 7 ☐ 8 ☐ 9 ☐ 10 Completely

SECTION 2: BE

3. I've been feeling optimistic about the future

☐ 1 - None of the time ☐ 2 - Rarely ☐ 3 - Some of the time ☐ 4 - Often ☐ 5 - Always

4. I've been feeling useful

☐ 1 - None of the time ☐ 2 - Rarely ☐ 3 - Some of the time ☐ 4 - Often ☐ 5 - Always

5. I’ve been feeling relaxed

- [ ] 1 - None of the time
- [ ] 2 - Rarely
- [ ] 3 - Some of the time
- [ ] 4 - Often
- [ ] 5 - Always

6. I’ve been dealing with problems well

- [ ] 1 - None of the time
- [ ] 2 - Rarely
- [ ] 3 - Some of the time
- [ ] 4 - Often
- [ ] 5 - Always

7. I’ve been thinking clearly

- [ ] 1 - None of the time
- [ ] 2 - Rarely
- [ ] 3 - Some of the time
- [ ] 4 - Often
- [ ] 5 - Always

8. I’ve been able to make up my own mind about things

- [ ] 1 - None of the time
- [ ] 2 - Rarely
- [ ] 3 - Some of the time
- [ ] 4 - Often
- [ ] 5 - Always

9. I’ve been feeling close to other people

- [ ] 1 - None of the time
- [ ] 2 - Rarely
- [ ] 3 - Some of the time
- [ ] 4 - Often
- [ ] 5 - Always

10. How often do you spend 30 minutes playing sports or physical exercise?

- [ ] 1 - Never/almost never
- [ ] 2 - Less than monthly
- [ ] 3 - 1-3 times a month
- [ ] 4 - 1-2 times a week
- [ ] 5 - Everyday/ almost everyday

11. How often do you spend 15 minutes walking or cycling?

- [ ] 1 - Never/almost never
- [ ] 2 - Less than monthly
- [ ] 3 - 1-3 times a month
- [ ] 4 - 1-2 times a week
- [ ] 5 - Everyday/ almost everyday

12. How often do you attend courses of some kind?

- [ ] 1 - Never/almost never
- [ ] 2 - Less than monthly
- [ ] 3 - 1-3 times a month
- [ ] 4 - 1-2 times a week
- [ ] 5 - Everyday/ almost everyday

13. How often do you spend time informally learning about something new?

- [ ] 1 - Never/almost never
- [ ] 2 - Less than monthly
- [ ] 3 - 1-3 times a month
- [ ] 4 - 1-2 times a week
- [ ] 5 - Everyday/ almost everyday

14. I notice and appreciate the little things in life

- ☐ 1 - Disagree strongly
- ☐ 2 - Disagree
- ☐ 3 - Neither
- ☐ 4 - Agree
- ☐ 5 - Agree strongly

15. I can laugh and see the funny side of things

- ☐ 1 - Disagree strongly
- ☐ 2 - Disagree
- ☐ 3 - Neither
- ☐ 4 - Agree
- ☐ 5 - Agree strongly

SECTION 4: CONNECT

16. How often do you meet socially with friends, relatives or work colleagues?

- ☐ 1 - Never/almost never
- ☐ 2 - Less than monthly
- ☐ 3 - 1-3 times a month
- ☐ 4 - 1-2 times a week
- ☐ 5 - Everyday/ almost everyday

17. How often do you participate in social activities of a club, society or an association?

- ☐ 1 - Never/almost never
- ☐ 2 - Less than monthly
- ☐ 3 - 1-3 times a month
- ☐ 4 - 1-2 times a week
- ☐ 5 - Everyday/ almost everyday

18. How often do you feel lonely?

- [ ] 1 - Always
- [ ] 2 - Often
- [ ] 3 - Sometimes
- [ ] 4 - Occaisionally
- [ ] 5 - Never

19. How often do you help out informally with friends or neighbours?

- [ ] 1 - Never/almost never
- [ ] 2 - Less than monthly
- [ ] 3 - 1-3 times a month
- [ ] 4 - 1-2 times a week
- [ ] 5 - Everyday/ almost everyday

20. How often do you get involved in work for voluntary or charitable organisations?

- [ ] 1 - Never/almost never
- [ ] 2 - Less than monthly
- [ ] 3 - 1-3 times a month
- [ ] 4 - 1-2 times a week
- [ ] 5 - Everyday/ almost everyday

21. Do you have a friend/s or family member/s with whom you can discuss personal matters?

- [ ] Yes
- [ ] No

22. I feel like I belong to this neighbourhood

- [ ] 1 - Disagree strongly
- [ ] 2 - Disagree
- [ ] 3 - Neither
- [ ] 4 - Agree
- [ ] 5 - Agree strongly

Part 2: Digital Technology

In this part of the survey, you will be asked eight different questions about your ability to use digital technology, your views on technology and how often you use technology.

When you answer these questions, please think in particular about video calls and messaging such as WhatsApp, Skype and Facebook Messenger.

1. How often do you use this type of technology?

- ☐ Several times a day
- ☐ Once a day
- ☐ Weekly
- ☐ Monthly
- ☐ Less than once a month
- ☐ Never
- ☐ I don’t know

2. To what extent do you agree with the following statements?	1 - Strongly disagree	2 - Disagree	3 - Neither agree nor disagree	4 - Agree	5 - Strongly agree
2a. I find this technology easy to use.	☐	☐	☐	☐	☐
2b. I find this technology useful in my everyday life.	☐	☐	☐	☐	☐
2c. I find this technology reliable in that it operates smoothly.	☐	☐	☐	☐	☐
2d. I am likely to use this type of technology in the next month.	☐	☐	☐	☐	☐
2e. I feel that I am able to use this digital technology independently, without any help from other people.	☐	☐	☐	☐	☐
2f. This digital technology helps me feel close to other people.	☐	☐	☐	☐	☐
2g. I want to use this technology because my friends are using this technology.	☐	☐	☐	☐	☐
2h. I want to use this technology because my family are using this technology.	☐	☐	☐	☐	☐
2i. The people who are most important to me think I should use this technology.	☐	☐	☐	☐	☐

**3. To what extent do you agree with the following statements?**	1 - Strongly disagree	2- Disagree	3 - Neither agree nor disagree	4 - Agree	5 - Strongly agree
**3a. I feel confident that I am able to use most types of digital technology to do the things that I want to do.**	☐	☐	☐	☐	☐
**3b. I enjoy using most types of digital technology.**	☐	☐	☐	☐	☐

4. How would you rate your ability to use the Internet?

☐ Excellent ☐ Good ☐ Fair ☐ Poor ☐ Very poor ☐ Don't know/ can't say

5. To what extent do you agree with the following statement? "The internet makes my life easier."

☐ 1 - Disagree strongly ☐ 2 - Disagree ☐ 3 - Neither ☐ 4 - Agree ☐ 5 - Agree strongly

6. How would you rate your ability to use smartphones?

☐ Excellent ☐ Good ☐ Fair ☐ Poor ☐ Very poor ☐ Don't know/ can't say

7. How often do you go online to find information about health-related issues or medical care?

- ☐ Several times a day
- ☐ Once a day
- ☐ Weekly
- ☐ Monthly
- ☐ Less than once a month
- ☐ Never
- ☐ I don't know

8. To what extent do you agree with the following statement? "I feel that, if I want to, there are people I can talk to online if I'm feeling lonely."

- ☐ 1 - Disagree strongly
- ☐ 2 - Disagree
- ☐ 3 - Neither
- ☐ 4 - Agree
- ☐ 5 - Agree strongly

SECTION 5: DEMOGRAPHICS

As researchers, we understand that different identities and social categories create overlapping and independent systems of discrimination and disadvantage. We are asking these important demographic questions because we must consider everything and anything that can marginalise people. Please answer the following questions to help us in our goal towards achieving fair opportunities for everyone.

1. Postcode

2. Gender

☐ Male ☐ Female ☐ Non-binary ☐ Prefer not to say

3. Age

☐ 18-24 ☐ 25-34 ☐ 35-44 ☐ 45-54 ☐ 55-64 ☐ 65-74 ☐ 75+ ☐ Prefer not to say

4. How would you describe your racial identity?

5. How would you describe your national or cultural identity?

6. Do you consider yourself to have a disability?

☐ Yes ☐ No ☐ Prefer not to say

Thank you

Thank you for completing this survey!

If you have any questions relating to the responses you have provided please contact the Smartline team on **smartlineresearch@exeter.ac.uk**
