# Supplementary material for: Telephone-Based Training Intervention for Using Digital Communication Technologies for Social Housing Residents During the COVID-19 Pandemic: Mixed Methods Feasibility and Acceptability Evaluation
Source: JMIR Form Res. 2024 Jan 26;8:e45506. doi: 10.2196/45506 (PMC10858426; doi:10.2196/45506)
Supplement: Multimedia Appendix 3 [file formative_v8i1e45506_app3.pdf]

- This information will also be kept until 31 December 2033 as required by the project funder. Your information will not be shared with any third party. You have a right to access your own data or for it to be deleted.
- If you have any concerns regarding the handling of your data or would like your consent withdrawn at any time, please contact the research team on **smartlineresearch@exeter.ac.uk** or Karen **07968 706114** or Phil **01209 200169**.

What if I change my mind about participation in the study?

You can withdraw your consent to sharing of information for research and evaluation purposes, with all or specific organisations, at any time and this will not affect the support you receive from Cornwall Council's Digital Inclusion Team.

Is there any incentive to participate?

Yes. The incentive for completion of the follow up survey (4 months after digital support) is a £10 shopping voucher which is accepted in most high street stores and online. All participants who take part in interviews will receive a £10 voucher.

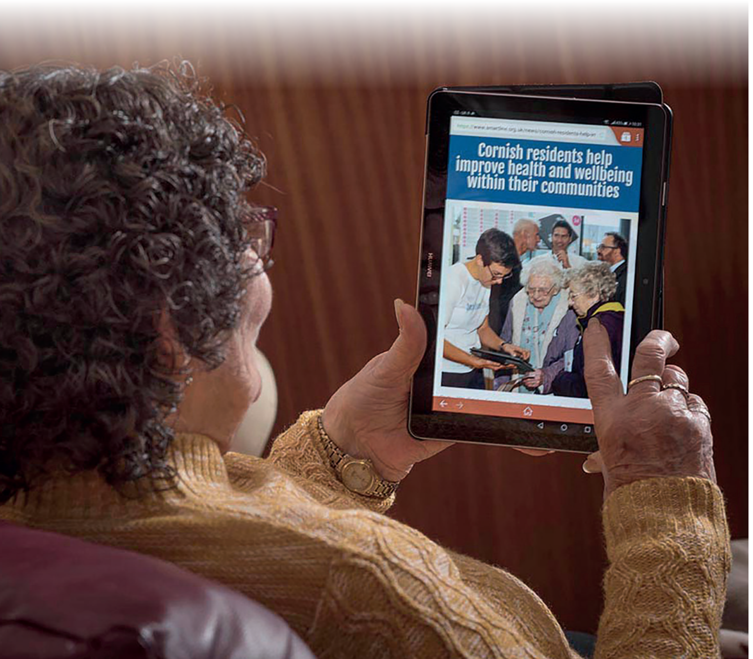

Who should I contact if I have any further questions?

If you have any questions about our project, either now or in the future, please feel free to contact:

Smartline Email: **smartlineresearch@exeter.ac.uk**

Philip Gilbert (Smartline Project Co-ordinator, Coastline) Telephone: **01209 200169**

Karen Spooner (Smartline Community Engagement Officer) Telephone: **07968 706114**

Dr. Tim Walker (Smartline Research Fellow, University of Exeter) Email: **t.w.walker@exeter.ac.uk**

Prof. Karyn Morrissey (Principal Investigator and Associate Professor, University of Exeter) Email: **k.morrissey@exeter.ac.uk**

This project has been reviewed and approved by the University of Exeter College of Life and Environmental Sciences Ethics Committee

Reference number: eCORN002229
Date: 08/09/2020

Smartline

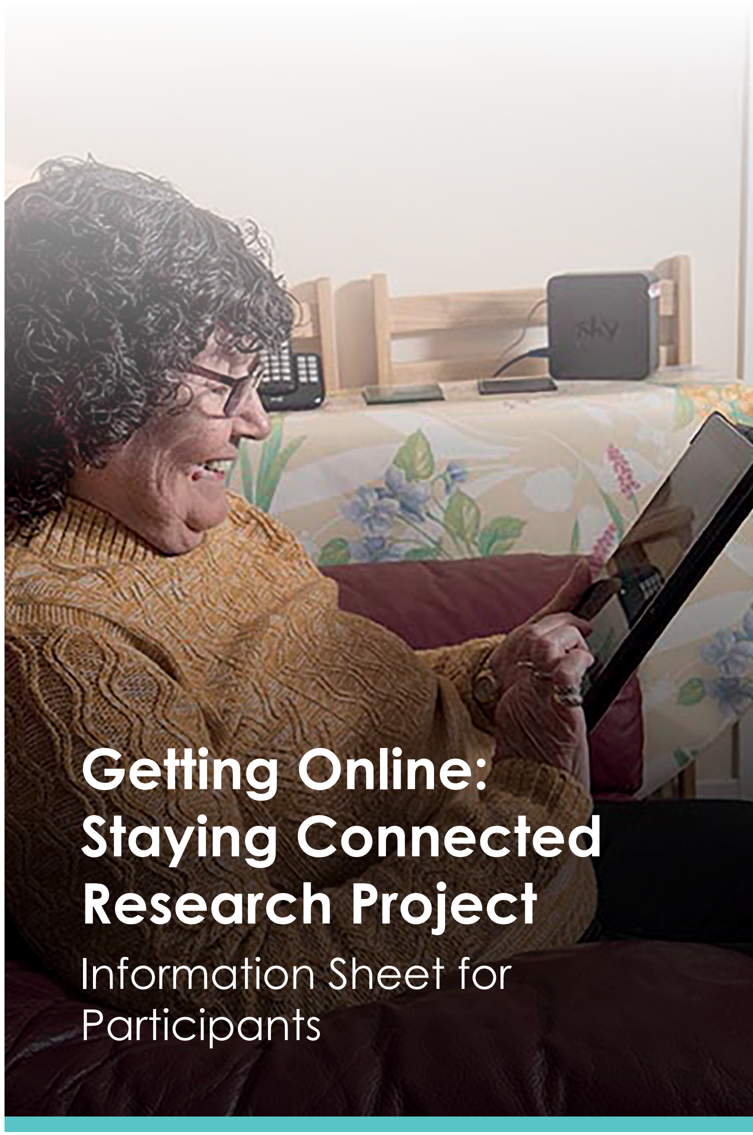

Getting Online: Staying Connected Research Project

Information Sheet for Participants

European Union
European Regional Development Fund

HM Government

Thank you for showing an interest in this project. Please read this information sheet carefully before deciding whether or not to take part.

What is Smartline?

- Smartline is a research project in partnership with the University of Exeter, Coastline Housing, Cornwall Council, Volunteer Cornwall and the South West Academic Health Science Network.
- Smartline is part funded by the European Regional Development Fund and Cornwall Council.
- Smartline is investigating how digital technology could help people live longer and healthier lives in stronger communities.
- Information about the Smartline project can be found online here: smartline.org.uk or if you would like to speak to us you can call Karen on **07968 706114**, or if you are a Coastline customer call Phil on **01209 200169.**
- Smartline is launching a project to understand how getting online and using digital technology can help people in Cornwall stay connected, become better informed and improve their wellbeing.

What is the Getting Online: Staying Connected Project?

- The Getting Online: Staying Connected Project seeks to understand the benefits of learning to use digital communications applications such as smartphones, social media apps, video calling and other features.
- We have partnered with Cornwall Council's Digital Inclusion Team to evaluate their digital support programme.
- The digital support programme is a service delivered by Cornwall Council which provides one-to-one telephone support to get people online and using online services with confidence. Helping people to use WhatsApp or Zoom for example.

What does participation in this study involve?

- Participation in this study would involve receiving telephone support from Cornwall Council's Digital Inclusion Team and completing surveys about your digital skills and general wellbeing. You will be asked to complete the survey before receiving the support and six months after.
- You may also be asked if you would like to be interviewed about your experiences. If you consent to take part in an interview, interviews will take place at a time that is convenient for you, by telephone. We will audio-record the interview with your permission (the recording will only be accessed by the research team for note-taking purposes).
- There might be a delay of up to 4 weeks between signing up to this project and receiving support.

How long will the digital training take?

The training is at your own pace. This might involve one phone call or several phone calls with printed help guides emailed to you.

Who can participate?

This study is open to all who are over the age of 18 years old.

Do I need anything to participate?

Yes, you will need an internet enabled device and access to an internet connection.

What will the surveys ask?

There are two parts to the survey. The first will ask about your physical, emotional, and social wellbeing. The second part will ask about your existing technology use, skills and views on technology.

How long will the surveys take?

You will be asked to complete the survey two times over the duration of the study: once before you receive the digital support and again 4 - 6 months after receiving the digital support. The survey takes approximately 20 minutes to complete.

How will my information be handled?

The University of Exeter processes personal data for the purposes of carrying out research in the public interest. The University will endeavour to be transparent about its processing of your personal data and this information sheet should provide a clear explanation of this. If you do have any queries about the University's processing of your personal data that cannot be resolved by the research team, further information may be obtained from the University's Data Protection Officer by emailing **dataprotection@exeter.ac.uk** or at **www.exeter.ac.uk/dataprotection.**

We will not collect or store any data without your consent. If you provide your consent your data will be handled in the following ways:

- After completing the wellbeing and digital skills survey, your information will be anonymised and processed and coded with study numbers so no names will be retained.
- The anonymised and processed data will be stored in a repository and kept until 31 December 2033 as required by the project funder.
- Only the research team will have access to a list containing identifiable information such as your name, address, phone number, study number, so we can contact you during the study if needed. This list will be securely stored on the University server with access limited to the core research team.
- Any printed copies of the anonymised wellbeing and digital skills surveys will be stored in a locked cupboard at University of Exeter Medical School, Knowledge Spa.
