# Supplementary material for: Telephone-Based Training Intervention for Using Digital Communication Technologies for Social Housing Residents During the COVID-19 Pandemic: Mixed Methods Feasibility and Acceptability Evaluation
Source: JMIR Form Res. 2024 Jan 26;8:e45506. doi: 10.2196/45506 (PMC10858426; doi:10.2196/45506)
Supplement: Multimedia Appendix 4 [file formative_v8i1e45506_app4.pdf]

GETTING ONLINE: STAYING CONNECTED

CONSENT FORM

Thank you for showing an interest in this project. Please read the Information Sheet Version Number [5] Dated [11.08.20] before deciding whether or not to participate. Please note that if you have any unanswered questions about this study then you should NOT complete this form but please contact us at **smartlineresearch@exeter.ac.uk** or by calling Phil Gilbert on **01209 200169** / Karen Spooner on **07968 706114** and we will try to help.

Please insert your initials to confirm you understand what the project involves and your consent:

		Initial in the boxes below against each question
1	I confirm I have read the Information Sheet concerning this project and understand what it is about.	
2	I confirm that I have had the opportunity to discuss the study with the Smartline research team. I do not have any further questions about this study.	
3	I understand that I am free to request further information at any stage.	
4	I understand that the information collected during this study will remain strictly confidential and accessible only to appropriate members of the research team.	
5	I have read and understood the privacy policy relating to the use of my data and I am content with how my data will be used.	
6	The data collected may be merged with other data held about my household or property by Coastline Housing, Cornwall Council and Volunteer Cornwall.<br>I give consent for this data merger.	
7	I understand the results of the project may be published but my anonymity will be preserved.	
8	I understand that my participation is voluntary and that I am free to withdraw without giving any reason.<br>I understand that if I withdraw from the study my data will be deleted.	

I agree to take part in this project.

.... (Printed name of participant)

.... (Signature of participant)

.... (Date)

The research team wish to do a follow up interviews with some study participants:

		Please tick	
9	I confirm I have read the Information Sheet concerning this project and understand what it is about.	Yes	No

This project has been reviewed and approved by the University of Exeter College of Life and Environmental Sciences Ethics Committee

Reference number: eCORN002229 Date: 08/09/2020

Coastline housing Volunteer Cornwall UNIVERSITY OF EXETER South West Academic Health Science Network one and all onen hag oll CORNWALL COUNCIL
