# Supplementary material for: Telephone-Based Training Intervention for Using Digital Communication Technologies for Social Housing Residents During the COVID-19 Pandemic: Mixed Methods Feasibility and Acceptability Evaluation
Source: JMIR Form Res. 2024 Jan 26;8:e45506. doi: 10.2196/45506 (PMC10858426; doi:10.2196/45506)
Supplement: Multimedia Appendix 5 [file formative_v8i1e45506_app5.docx]

| **Basic Digital Skills** |
| --- |
| Lesson Content Guidance |
| November 2018 |
| Digital Inclusion Team |

| **Date issued** | **Date Effective** | **Procedure ID** | **Issue No.** | **Updated by** |
| --- | --- | --- | --- | --- |
| 30^th^ November 2018 | 30^th^ November 2018 | DIO 005 | 001 | Dawn Stoddern |

**Introduction and purpose**

The goal of a Basic Digital Skills course is to equip learners with computer skills as defined by Tech Partnership – Basic Digital Skills Framework.

Beginners who have very little experience with computers can learn the basics on this course. Fundamental operations and functions of the computer will be covered. Specific topics include the use of the Internet, basic computer functions and safety.

The purpose of this document is to clearly set out the content needed to deliver a Basic Digital Skills course delivered by the Digital and Online Support Team. This guidance intends to ensure consistency and to embed best practice.

Week 1

Alongside introducing and practicing the Digital Foundation Skills all adults need, this session will specifically focus on

- Problem solving – Finding solutions to problems using digital tools and online services

| 1. **Turn on a device** 2. **Use the available controls** 3. **Make use of accessibility tools to make it easier to use** 4. **Interact with home screen** 5. **Understand that the internet allows access to information and content and that it will connect through WiFi** 6. **Connect to a safe and secure WiFi** 7. **Open a browser to find and use websites** 8. **Know that passwords and personal information need to safe** 9. **Can update and change password when prompted to do so** | 1. **use the internet to find information that helps to solve problems** 2. **use the internet to find sources of help for a range of activities** 3. **use chat facilities to help solve problems** 4. **use online tutorials FAQs and advice forums to solve problems and improve skills in using devices software and applications** |
| --- | --- |

**Life Examples**

Use the internet to find specific information related to life tasks that need to be carried out: e.g. finding a recipe or finding information that helps plan travel

Use the help, FAQ section or chat facility of a manufacturer’s website or other related content to work out how to fix an issue with a device.

Find out how to do something by using a tutorial video such as those found on You Tube

Week 2

Alongside introducing and practicing the Digital Foundation Skills all adults need, this session will specifically focus on

- Communicating- Using tools and online services to communicate, collaborate and share

| **Digital Foundation Skills** | **Communicating** |
| --- | --- |
| 1. **Turn on a device** 2. **Use the available controls (keyboard, Mouse, touchscreen)** 3. **Make use of accessibility tools to make it easier to use – Use settings menus to change display and make content easier to read** 4. **Interact with home screen – choose correct icons** 5. **Understand that the internet allows access to information and content and that it will connect through WiFi – use WiFi settings and insert password** 6. **Connect to a safe and secure WiFi** 7. **Open a browser to find and use websites – locate bowser icon and locate website** 8. **Know that passwords and personal information need to safe** 9. **Can update and change password when prompted to do so – keep information safe not shared or written down** | 1. **Understand the importance of communicating safely** 2. **Can set up an email account** 3. **Can communicate with others using email and other messaging apps** 4. **Use word processing applications to create documents** 5. **Communicate using video tools** 6. **Can post messages, photographs, videos or blogs on social media platforms** |

**Life examples**

Send photographs and other documents to friends and family as an email attachment.

Set up and use video telephony products such as Facetime or Skype for video communications with friends and family

Post appropriately on social media visit and post for forums such as Musnet or Reddit

Week 3

Alongside introducing and practicing the Digital Foundation Skills all adults need, this session will specifically focus on

| **Digital Foundation Skills** | **Transacting** |
| --- | --- |
| 1. **Turn on a device** 2. **Use the available controls** 3. **Make use of accessibility tools to make it easier to use** 4. **Interact with home screen** 5. **Understand that the internet allows access to information and content and that it will connect through WiFi** 6. **Connect to a safe and secure WiFi** 7. **Open a browser to find and use websites** 8. **Know that passwords and personal information need to safe** 9. **Can update and change password when prompted to do so** | 1. **Can set up an account online using appropriate websites and apps to buy goods and services** 2. **Can access and use public services online including filling in form** 3. **Can use different payment systems such as credit/debit card, direct bank transfer, and phone accounts to make payments** 4. **Can upload documents and photographs when this required to complete an online transaction** 5. **Can fill in online forms when required to complete an online transaction Can manage money and transactions online and securely through bank websites and apps** |

- Transacting – Registering and applying for services, buy and sell goods and services, and administer and manage transactions online

**Life examples**

Set up online accounts for public services such as with local council or a Government Department

Set up online accounts with retailers to order and pay for goods online such as Amazon or eBay

Use travel websites and Apps to book tickets and make reservations

Make a GP appointment online

Complete online forms to apply for a television license or road tax

Set up and use online banking through websites or Apps keeping access information secure

Upload a CV to an online recruitment site

Week 4

Alongside introducing and practicing the Digital Foundation Skills all adults in this session will specifically focus on

- Handling Information and Content – Find manage and store digital information and content securely.

| **Digital Foundation Skills** | **Information and Content** |
| --- | --- |
| 1. **Turn on a device** 2. **Use the available controls** 3. **Make use of accessibility tools to make it easier to use** 4. **Interact with home screen** 5. **Understand that the internet allows access to information and content and that it will connect through WiFi** 6. **Connect to a safe and secure WiFi** 7. **Open a browser to find and use websites** 8. **Know that passwords and personal information need to safe** 9. **Can update and change password when prompted to do so** | 1. **Understand not all online information and content is reliable** 2. **Can evaluate what information or content may or may not be reliable** 3. **Can use bookmarks to save and retrieve information on web browser** 4. **Can access information and content from different devices** 5. **Understand that the cloud is a way to store information and content in a remote location** 6. **Can organise information and content using files and folders on my device or in the cloud** 7. **Can use the internet legally to access content for entertainment including games films and music** |

**Life examples**

Understand that not all entries in online encyclopaedias such as Wikipedia are true and reliable

Search for news using a browser such as Chrome, Internet Explorer or Safari

Use a cloud storage account for music or photo collection (from legal sources such as Apple iCloud Instagram) and access the collections from different devices such as laptop or a smartphone

Stream music from legal sites such a Spotify or Apple music or watch streamed movies from legal sources such as Netflix or Amazon

**Monitoring arrangements**

Each Digital Inclusion Officer will be responsible for using this guidance to design lesson plans for Basic Digital Skills courses.

**Corporate and legal**

*Data Protection Act 2018*,

GDPR 2018

*Health and Safety legislation*,

*Disability and Discrimination Act*

**Prepared by**: Dawn Stoddern Digital Inclusion Team Lead
