# Supplementary material for: Telephone-Based Training Intervention for Using Digital Communication Technologies for Social Housing Residents During the COVID-19 Pandemic: Mixed Methods Feasibility and Acceptability Evaluation
Source: JMIR Form Res. 2024 Jan 26;8:e45506. doi: 10.2196/45506 (PMC10858426; doi:10.2196/45506)
Supplement: Multimedia Appendix 6 [file formative_v8i1e45506_app6.docx]

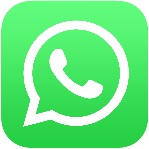
How To Install WhatsApp

1. To install the app, tap
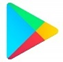
**Google Play** app (Android) or


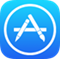
**App store** (Apple) icon


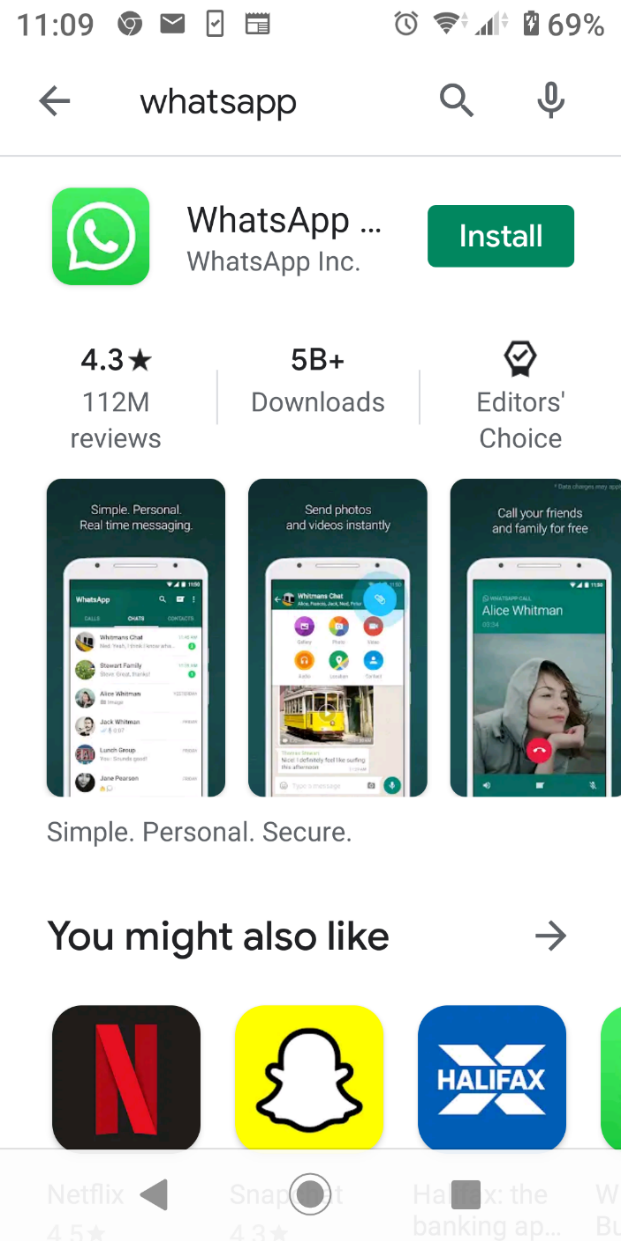


1. Search for **WhatsApp** and tap **Install**


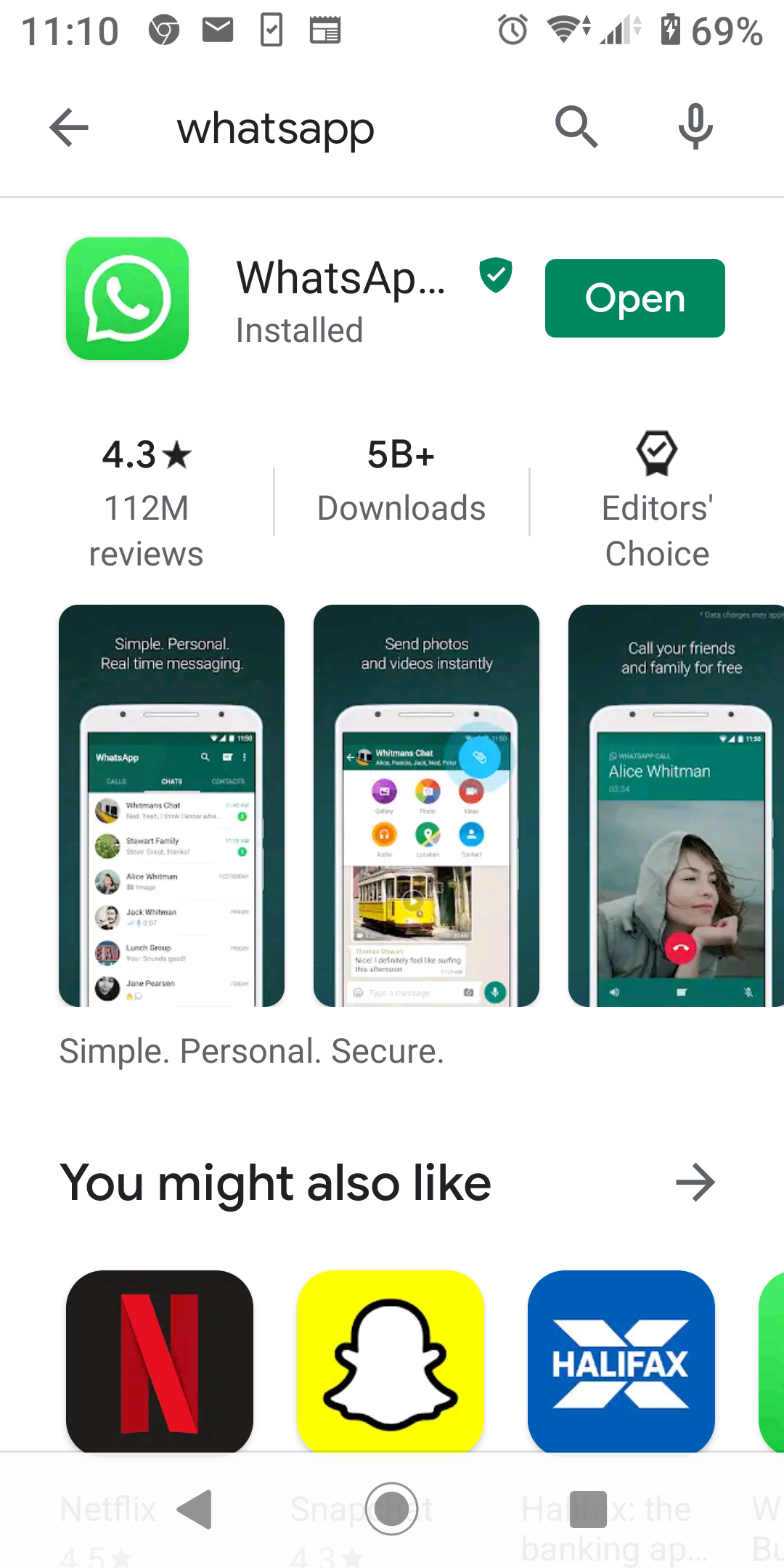


1. When it is installed tap **Open**


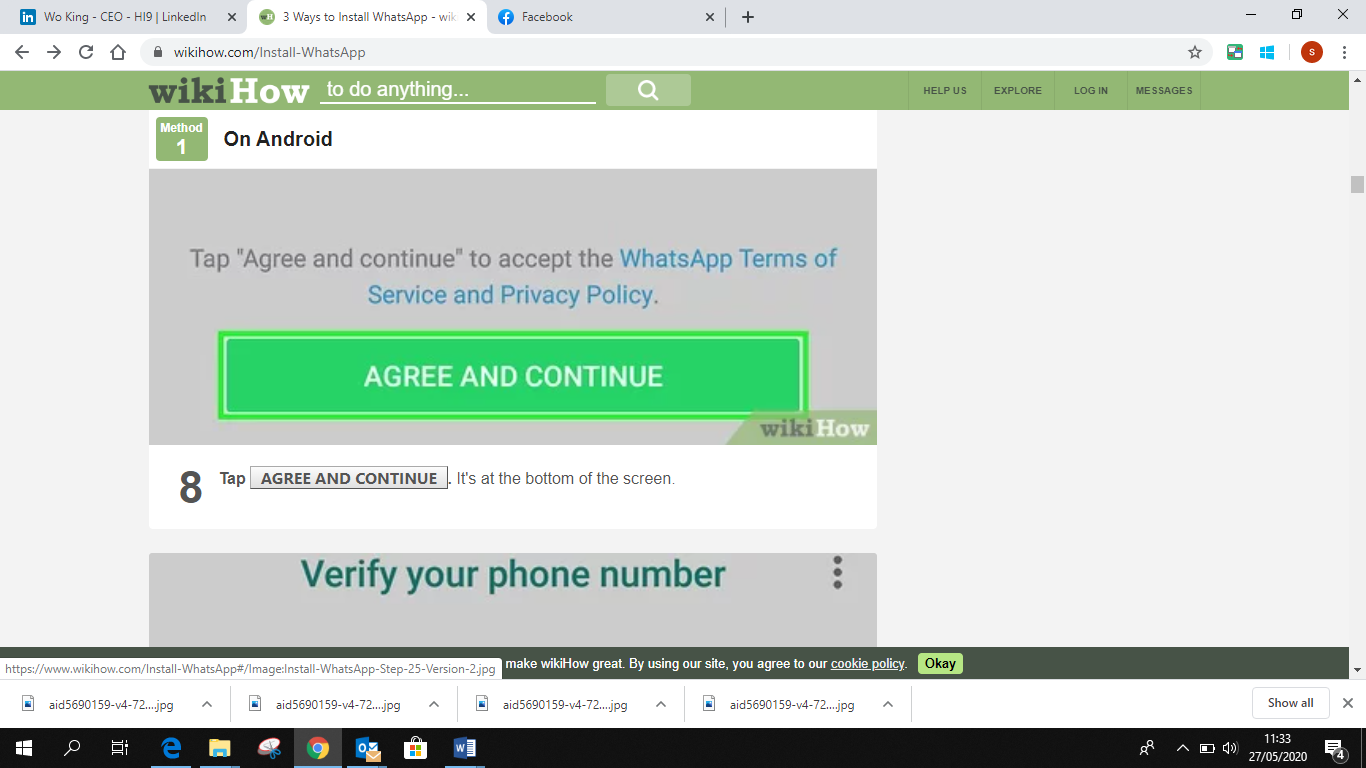


1. **Agree** the Terms and Conditions


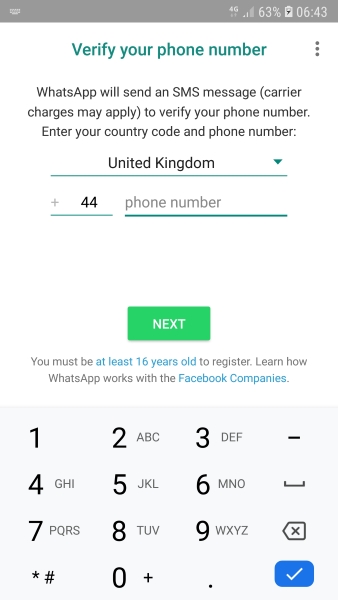


1. Select **United Kingdom** from the drop down

then **type your phone number** and tap **Next**


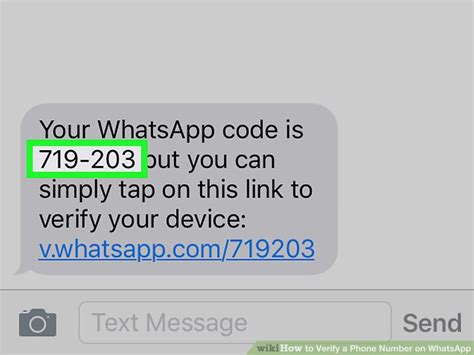


1. You will receive a **text message** with a

6 digit code**, tap the blue link**


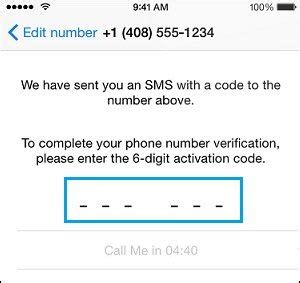


1. **Type the six-digit code** into WhatsApp


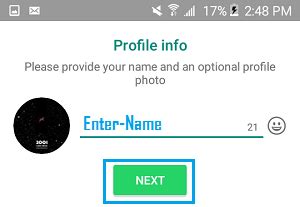


1. Enter your **name** and a **photo**, photo is optional

Finally tap **Next**
