# Supplementary material for: Telephone-Based Training Intervention for Using Digital Communication Technologies for Social Housing Residents During the COVID-19 Pandemic: Mixed Methods Feasibility and Acceptability Evaluation
Source: JMIR Form Res. 2024 Jan 26;8:e45506. doi: 10.2196/45506 (PMC10858426; doi:10.2196/45506)
Supplement: Multimedia Appendix 7 [file formative_v8i1e45506_app7.docx]

File E: Theoretical basis of questions in detail with references to support content validity

| **Outcome being assessed** | **Validated?** | **Question** | **Source** | **Theory (SDT/TAM/UTAUT/TPB)**  **and concept** | **References (relevant theories, question sources and any studies that have used the question)** |
| --- | --- | --- | --- | --- | --- |
| Competence: Perceived ease of use of specified digital technology | N | I find this technology easy to use.  *[1 – Strongly disagree]*  *[2 – Disagree]*  *[3 – Neither agree nor disagree]*  *[4 – Agree]*  *[5 – Strongly agree]* | Our own theory-based question (based on TAM and usability scales) | SDT (competence)  TAM (ease of use)  UTAUT (effort expectancy) | Reference SDT (Deci and Ryan 2000 [50])  Reference TAM (Davis, 1989 [56])  Reference UTAUT (Venkatesh et al 2003 [51])  Reference UMUX (Finstad, 2010 [58])    TAM – See Constantinides et al (2013) [55] Table 1 below for list of studies which provide “content validity”    This question is also same/similar to item 3 in the UMUX, except UMUX uses a 7 point scale. |
| Perceived usefulness of specified digital technology | N | I find this technology useful in my everyday life.  *[1 – Strongly disagree]*  *[2 – Disagree]*  *[3 – Neither agree nor disagree]*  *[4 – Agree]*  *[5 – Strongly agree]* | Our own theory-based question (based on TAM and usability scales) | SDT (motivation)  TAM (perceived usefulness)  UTAUT (performance expectancy)  TPB (attitude) | Reference SDT (Deci and Ryan 2000 [50])  Reference TAM (Davis, 1989 [56])  Reference UTAUT (Venkatesh et al., 2003 [51])  Reference TPB (Ajzen 1991 [49])    TAM – See Constantinides et al (2013) [55] Table 1 below for list of studies which provide “content validity” |
| Perceived reliability of specified digital technology | N | I find this technology reliable in that it operates smoothly.  *[1 – Strongly disagree]*  *[2 – Disagree]*  *[3 – Neither agree nor disagree]*  *[4 – Agree]*  *[5 – Strongly agree]* | Our own theory-based question | TAM (ease of use)  UTAUT (facilitating conditions) | Reference TAM (Davis, 1989 [56])  Reference UTAUT (Venkatesh et al., 2003 [51]) |
| Intentions to continue using the specific digital technology | N | I am likely to use this type of technology in the next month.  *[1 – Strongly disagree]*  *[2 – Disagree]*  *[3 – Neither agree nor disagree]*  *[4 – Agree]*  *[5 – Strongly agree]* | Our own theory-based question | SDT (motivation/engagement)  TAM/UTAUT/TPB (intentions) | Reference SDT (Deci and Ryan 2000 [50])  Reference TAM (Davis, 1989 [56])  Reference UTAUT (Venkatesh et al., 2003 [51])  Reference TPB (Ajzen 1991 [49])    This question has vague similarity (with regard to frequency of use) with item 1 of the System Usability Scale (SUS) (Brooke, 1996 [54]). “I think I would like to use this system frequently”, strongly disagree to strongly agree, 1-5 scale (as is ours).    Also closely related to the ‘intention to use’ items in the Constantinides et al (2013) [55] surveys (see Constantinides table 1 below for studies that have used these items).  E.g. IU3: “I intend to begin or continue using [‘X’ technology]”, 1-5 scale strongly disagree to strongly agree. |
| Competence: Autonomy  (self-perceived autonomy relating to specified technology use) | N | I feel that I am able to use this digital technology independently, without any help from other people.  *[1 – Strongly disagree]*  *[2 – Disagree]*  *[3 – Neither agree nor disagree]*  *[4 – Agree]*  *[5 – Strongly agree]* | Our own theory-based question | SDT (autonomy)  TPB (perceived behavioural control) | Reference SDT (Deci and Ryan 2000 [50])  Reference TPB (Ajzen 1991 [49]) |
| Relatedness  (social connectedness resulting from specified technology use) | N (adapted from Happiness Pulse relationships question: “I have been feeling close to other people” but with different response options). The Happiness Pulse question and the question it is based on from SWEMWBS are well validated. | This digital technology helps me feel close to other people.  *[1 – Strongly disagree]*  *[2 – Disagree]*  *[3 – Neither agree nor disagree]*  *[4 – Agree]*  *[5 – Strongly agree]* | Adapted from Happiness Pulse ‘relationships’ question (original source: SWEMWBS) | SDT (relatedness) | Happiness Pulse has been used with a nationally representative sample of 2,000 responses (collected by YouGov in October 2018) (Centre for Thriving Places 2020 [47,53]).    The Warwick-Edinburgh Mental Well-being scale (WEMWBS) [61] has been well validated. Original reference (Tennant, Hiller et al. 2007 [62]). The short version (SWEMWBS) has been validated in multiple countries and populations, including in the general community in four European countries (Koushede et al. 2019 [60]). |
| Subjective norms related to specified technology: friends (social influence – descriptive norms) | N | I want to use this technology because my friends are using this technology.  *[1 – Strongly disagree]*  *[2 – Disagree]*  *[3 – Neither agree nor disagree]*  *[4 – Agree]*  *[5 – Strongly agree]* | Our own theory-based question | UTAUT (social influence)  TPB (subjective norms) | Reference UTAUT (Venkatesh et al., 2003 [51])  Reference TPB (Ajzen 1991 [49]) |
| Subjective norms related to specified technology: family (social influence – descriptive norms) | N | I want to use this technology because my family are using this technology.  *[1 – Strongly disagree]*  *[2 – Disagree]*  *[3 – Neither agree nor disagree]*  *[4 – Agree]*  *[5 – Strongly agree]* | Our own theory-based question | UTAUT (social influence)  TPB (subjective norms) | Reference UTAUT (Venkatesh et al., 2003 [51])  Reference TPB (Ajzen 1991 [49]) |
| Subjective norms related to specified technology: influential others (social influence – injunctive norms) | N | The people who are most important to me think I should use this technology.  *[1 – Strongly disagree]*  *[2 – Disagree]*  *[3 – Neither agree nor disagree]*  *[4 – Agree]*  *[5 – Strongly agree]* | Our own theory-based question | UTAUT (social influence)  TPB (subjective norms) | Reference UTAUT (Venkatesh et al., 2003 [51])  Reference TPB (Ajzen 1991 [49]) |
| Frequency of using specified digital technology | Y (but adapted for Digital Inclusion Evaluation Toolkit.  Original question: “How often do you use the Internet?”). Response options are the same | How often do you use this type of technology?  *[Several times a day]*  *[Daily]*  *[Weekly]*  *[Monthly]*  *[Less than once a month]*  *[Never]*  *[Don’t know]* | Question from ‘regular internet use’ section in **Digital Inclusion Evaluation Toolkit ‘Digital’ domain.**  Adapted from original source: DCMS/OII standardised measures | SDT (behaviour)  TAM/UTAUT (actual use)  TPB (behaviour) | Digital Inclusion Evaluation Toolkit (Just Economics Research Ltd. 2017a [52]) and bank of outcomes (Just Economics Research Ltd. 2017b [59])    *See more information on the toolkit below the table.*    Original question has been used in DCMS/OII surveys as a standardised measure for assessing internet use. Nationally representative England sample. (Ipsos MORI Social Research Institute 2019 [59])    “How often do you complete a form or an application for something on your mobile phone” (M7, adults-media-use-and-attitudes survey, Ofcom 2022 [63]) |
| Confidence / self-efficacy relating to technology in general | N | I feel confident that I am able to use **most types** of digital technology to do the things that I want to do.  *[1 – Strongly disagree]*  *[2 – Disagree]*  *[3 – Neither agree nor disagree]*  *[4 – Agree]*  *[5 – Strongly agree]* | Our own theory-based question | SDT (competence) | Reference SDT (Deci and Ryan 2000 [50]) |
| Affective attitude / intrinsic motivation for digital technology use in general | N | I enjoy using **most types** of digital technology.  *[1 – Strongly disagree]*  *[2 – Disagree]*  *[3 – Neither agree nor disagree]*  *[4 – Agree]*  *[5 – Strongly agree]* | Our own theory-based question | SDT (motivation)  TAM (attitude)  TPB (attitude) | Reference SDT (Deci and Ryan 2000 [50])  Reference TAM (Davis, 1989 [56])  Reference TPB (Ajzen 1991 [49]) |
| Competence: Internet skills: Self-rating of ability to use the internet | Y | How would you rate your ability to use the Internet?  *[Excellent]*  *[Good]*  *[Fair]*  *[Poor]*  *[Bad]*  *[Don’t know/Can’t say]* | Question from ‘internet skills’ section in **Digital Inclusion Evaluation Toolkit ‘Digital’ domain.** Original source: DCMS/OII standardised measures | SDT (competence) | Digital Inclusion Evaluation Toolkit (Just Economics Research Ltd. 2017a [52]) and bank of outcomes (Just Economics Research Ltd. 2017b [59])    *See more information on the toolkit below the table.*    Original question has been used in DCMS/OII surveys as a standardised measure for assessing internet use. Nationally representative England sample. (Ipsos MORI Social Research Institute 2019 [59])    “Overall, how confident are you as an internet user?”  Very confident, Fairly confident, Neither confident nor not confident, Not very confident, Not at all confident, Don’t know (IN11A, adults-media-use-and-attitudes survey, Ofcom 2022 [63]) |
| Confidence, value and motivation (in relation to going online and the Internet) | Y | The Internet makes my life easier.  *[1 – Strongly disagree]*  *[2 – Disagree]*  *[3 – Neither agree nor disagree]*  *[4 – Agree]*  *[5 – Strongly agree]*  *[6 - Don’t know]* | **Digital Inclusion Evaluation Toolkit ‘Digital’ domain.** Original source: DCMS/OII standardised measures) | SDT (motivation)  TAM (attitude)  TPB (attitude) | Digital Inclusion Evaluation Toolkit (Just Economics Research Ltd. 2017a [52]) and bank of outcomes (Just Economics Research Ltd. 2017b [59])    *See more information on the toolkit below the table.*    Original question has been used in DCMS/OII surveys as a standardised measure for assessing internet use. Nationally representative England sample. (Ipsos MORI Social Research Institute 2019 [59]) |
| Competence: Smartphone skills: Self-rating of ability to use smartphones | Y (but adapted to ask about smart phones, original question: “How would you rate your ability to use the Internet?”) | How would you rate your ability to use smartphones?  *[Excellent]*  *[Good]*  *[Fair]*  *[Poor]*  *[Bad]*  *[Don’t know/Can’t say]* | Question from ‘internet skills’ section in **Digital Inclusion Evaluation Toolkit ‘Digital’ domain**. Adapted from DCMS/OII standardised measures. | SDT (competence) | Digital Inclusion Evaluation Toolkit (Just Economics Research Ltd. 2017a [52]) and bank of outcomes (Just Economics Research Ltd. 2017b [59])    *See more information on the toolkit below the table.*    Original question has been used in DCMS/OII surveys as a standardised measure for assessing internet use. Nationally representative England sample. (Ipsos MORI Social Research Institute 2019 [59]) |
| Proportion seeking health information online | Y (but adapted for Digital Inclusion Evaluation Toolkit) | How often do you go online to find information about health-related issues or medical care?  *[Several times a day]*  *[Daily]*  *[Weekly]*  *[Monthly]*  *[Less than monthly]*  *[Never]*  *[Don’t know]* | Question in **Digital Inclusion Evaluation Toolkit ‘Health’ domain.** Adapted from OxIS survey. | SDT (behaviour)  TAM/UTAUT (actual use) | Digital Inclusion Evaluation Toolkit (Just Economics Research Ltd. 2017a [52]) and bank of outcomes (Just Economics Research Ltd. 2017b [59])    *See more information on the toolkit below the table.*    OxIS survey report 2013 – question on information seeking online asked in 2005, 2007, 2009, 2011 and 2013 surveys (probably also in the 2019 survey but I couldn’t find this in the report) (Dutton, Blank et al. 2013 [57])    *See more information on OxIS below the table.* |
| Communicating and connecting with others online: Proportion reporting that they feel less socially isolated as a result of going online | Y? | I feel that, if I want to, there are people I can talk to online if I’m feeling lonely.  *[1 – Strongly disagree]*  *[2 – Disagree]*  *[3 – Neither agree nor disagree]*  *[4 – Agree]*  *[5 – Strongly agree]*  *[6 - Don’t know]* | Question in **Digital Inclusion Evaluation Toolkit ‘Socialising’ domain.** | SDT (relatedness) | Digital Inclusion Evaluation Toolkit (Just Economics Research Ltd. 2017a [52]) and bank of outcomes (Just Economics Research Ltd. 2017b [59])    *See more information on the toolkit below the table.* |

SDT = Self-Determination Theory

TAM = Technology Acceptance Model

TPB = Theory of Planned Behaviour

UTAUT = Unified Theory of Acceptance and Use of Technology

CSUQ, SUS, and UMUX = Usability Scales
